# Combining test statistics and models in bootstrapped model rejection: it is a balancing act

**DOI:** 10.1186/1752-0509-8-46

**Published:** 2014-04-17

**Authors:** Rikard Johansson, Peter Strålfors, Gunnar Cedersund

**Affiliations:** 1 Department of Biomedical Engineering (IMT), Linköping University, Linköping, Sweden; 2 Department of Clinical and Experimental Medicine (IKE), Linköping University, Linköping, Sweden

**Keywords:** Model rejection, Bootstrapping, Combining information, 2D, Insulin signaling, Model Mimicry, Likelihood ratio

## Abstract

**Background:**

Model rejections lie at the heart of systems biology, since they provide conclusive statements: that the corresponding mechanistic assumptions do not serve as valid explanations for the experimental data. Rejections are usually done using *e*.*g*. the chi-square test (*χ*^2^) or the Durbin-Watson test (DW). Analytical formulas for the corresponding distributions rely on assumptions that typically are not fulfilled. This problem is partly alleviated by the usage of bootstrapping, a computationally heavy approach to calculate an empirical distribution. Bootstrapping also allows for a natural extension to estimation of joint distributions, but this feature has so far been little exploited.

**Results:**

We herein show that simplistic combinations of bootstrapped tests, like the *max* or *min* of the individual p-values, give inconsistent, *i*.*e*. overly conservative or liberal, results. A new two-dimensional (2D) approach based on parametric bootstrapping, on the other hand, is found both consistent and with a higher power than the individual tests, when tested on static and dynamic examples where the truth is known. In the same examples, the most superior test is a 2D *χ*^2^*vsχ*^2^, where the second *χ*^2^-value comes from an additional help model, and its ability to describe bootstraps from the tested model. This superiority is lost if the help model is too simple, or too flexible. If a useful help model is found, the most powerful approach is the bootstrapped log-likelihood ratio (LHR). We show that this is because the LHR is one-dimensional, because the second dimension comes at a cost, and because LHR has retained most of the crucial information in the 2D distribution. These approaches statistically resolve a previously published rejection example for the first time.

**Conclusions:**

We have shown how to, and how not to, combine tests in a bootstrap setting, when the combination is advantageous, and when it is advantageous to include a second model. These results also provide a deeper insight into the original motivation for formulating the LHR, for the more general setting of nonlinear and non-nested models. These insights are valuable in cases when accuracy and power, rather than computational speed, are prioritized.

## Background

A key tool in systems biology is mathematical modeling [1]. Modeling allows for a more complete analysis of the true relationship between experimental data and possible mechanistic explanations, compared to what is feasible using only classical biochemical reasoning. Nevertheless, because the data are limited and the systems are highly complex, and because many of the model parameters have to be estimated and cannot be uniquely determined, drawing mechanistic conclusions from modeling is challenging. For instance, it is hard to produce validated models, or to find core predictions, *i*.*e*. model predictions with low uncertainty [2-4]. While model validation, in the strict sense, is not possible [5], model rejection and hypothesis testing are possible and highly useful applications in modeling, also for biological research [2,3,6-13].

Formally, model rejection methods evaluate whether the null hypothesis, $H_{0}$, that a specific model has generated some given data can be rejected or not. One common way to do this is to test whether the residuals, *i*.*e*. the differences between the simulated and measured data points, are too big. This can be checked using the *χ*^2^-test statistic. Alternatively, one might also wish to check whether the residuals are too correlated. This can be done using the whiteness test or the Durbin-Watson (DW) test [2,14]. However, there is a problem. These tests are dependent on analytical derivations for the distributions of the test statistic under $H_{0}$, but these derivations are based on a number of assumptions, which might not be fulfilled [2]. For instance, some commonly used assumptions are that the experimental noise is normally or log-normally distributed, that the parameter estimates have converged, and that the parameters appear linearly in the model [15-18]. Because many of these assumptions are unfulfilled in systems biology problems, it is problematic to use these analytical expression. Some of the reasons why the assumptions often are unfulfilled include that the availability of data in systems biology examples often is severely limiting, that the signal-to-noise ratio is poor, that the number of parameters that appears non-linearly and/or are unidentifiable often are high, and, for model comparison approaches, such as the likelihood ratio test, that the tested models are not nested [18-24]. For more information on these assumptions and limitations, we refer the reader to our previous paper [2].

To help overcome the problem of unfulfilled assumptions, one may try to replace the analytical expressions with empirical distributions of the test statistics. One way to derive the empirical distributions is to use bootstrap approaches. In general, bootstrap samples are artificially generated data sets, where the distribution of the bootstrap samples should reflect the variability of the data. Although most mathematical proofs for bootstrap approaches usually also are derived under asymptotic conditions, an almost asymptotic setting is often achieved already for moderate sample sizes. There are two types of bootstrap approaches: parametric and non-parametric [25-27]. Non-parametric bootstrap samples are generated from the original data set by drawing with replacement. Parametric bootstrap samples are generated from a specific model, *e*.*g*. an error model, that also incorporates some null-hypothesis about the underlying system. There is a rich literature for both parametric and non-parametric methods and their applications to statistical testing in biology [9-11,28-33].

**Figure 1:**
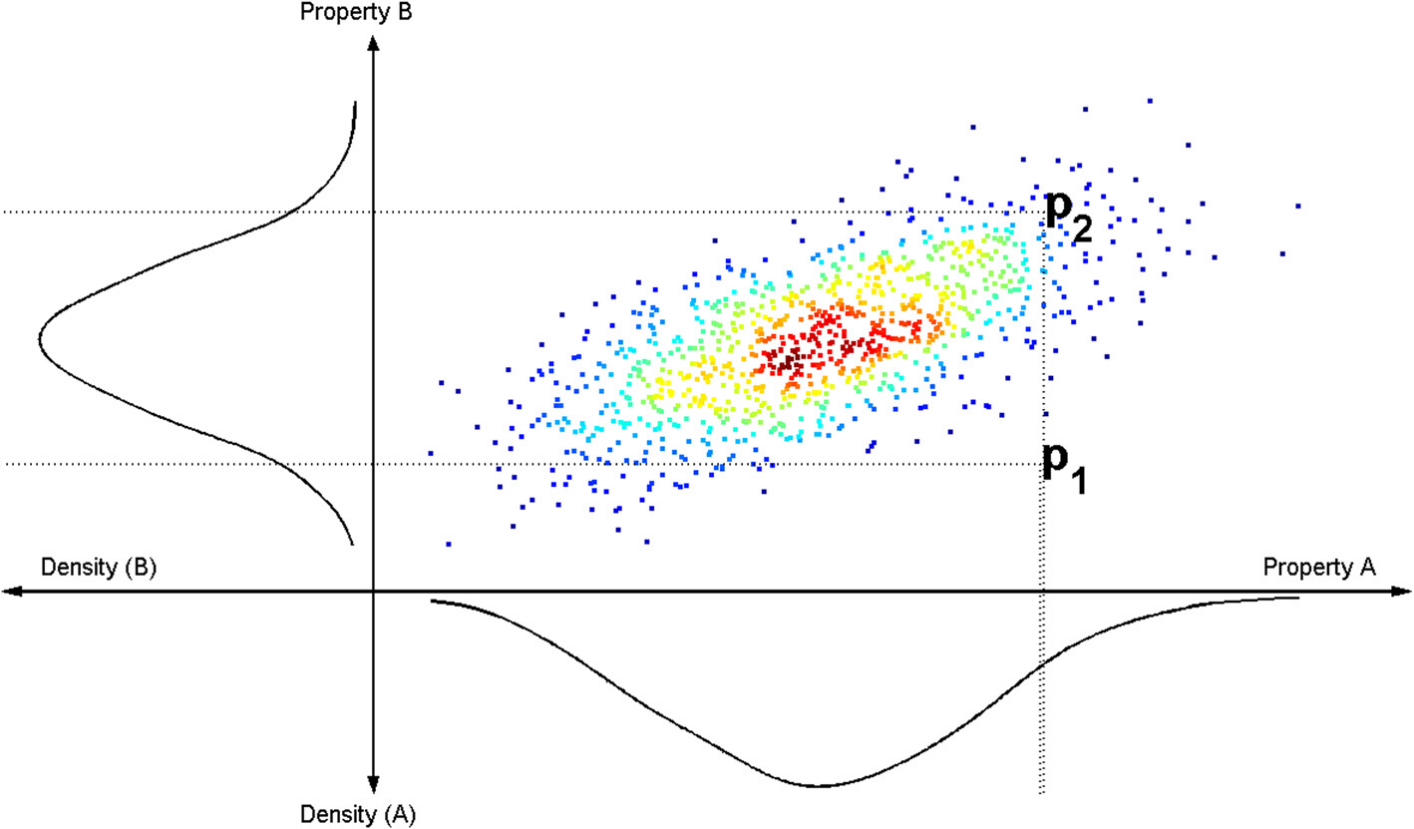
**Symbolic illustration of the advantage with the new herein presented 2D approach to combining test statistics.** The values of the two test statistics, **A** and **B**, are plotted on the positive x- and y-axes, respectively. The points correspond to bootstrap samples of pairs of these values, and the color of the cloud represent the probability density at the point: red means high density, *i*.*e*. a high probability to find a point there, and blue low. The 1D projections of the cloud are plotted on the negative x- and y-axes. The difference between a 1D analysis of these tests, considered independently, and the herein considered 2D approach, is found by comparing the two points *p*_1_ and *p*_2_. These two points correspond to two different hypothetical pairs of (A,B)-values, as calculated from the original data. If such a data point lies sufficiently outside the empirical distribution, the null hypothesis used to generate the empirical distribution is rejected. As can be seen, both *p*_1_ and *p*_2_ lies within the 1D distributions, and have essentially the same p-values, if the tests are two-sided. This stands in stark contrast to the situation in 2D: there *p*_2_ lies within the cloud, but *p*_1_ lies clearly outside. For this reason, the observation *p*_1_ would only be rejected in a 2D analysis, and not in a 1D analysis. Note that the main reason for this 2D advantage to be exploited is both that the 2D cloud does not lie parallel to either of the axes, and that the considered point just like *p*_1_ lies in a place that exploits the thinly populated areas that only are revealed in 2D.

A specific but seemingly unexplored advantage of using a bootstrap setting is that it allows for the natural combination of different test statistics (Figure 1). This advantage comes because, using bootstrapping, such combined statistical distributions can be calculated empirically, whereas the combination of such distributions analytically largely remains an unresolved problem. There is a field that deals with the combination of information (CI) [34], but this field primarily deals with combinations off different data sources, as in meta-analysis. For the combination of different statistical tests, one approach that has been considered is to combine the p-values [35-37]. There are some straightforward simplistic ways in which you could do these combinations. For instance, given two tests statistics, $T_{A}$ and $T_{B}$, for a specific model and data set, one could look at the maximum or minimum of the respective p-values etc. [34,37]. This corresponds to the principle of rejecting only if both tests reject, or if at least one of them rejects, respectively. However, there is a need to evaluate such naive combinations, in general and in the context of systems biology, and to provide more refined alternatives.

In this paper we examine how one could, should, and should not combine test statistics using parametric bootstrapping. The analysis shows that such above simplistic strategies to combine p-values are unsound, *i*.*e*. the tests are overly conservative or liberal, and in order to avoid this issue, one can instead use our proposed joint two-dimensional distribution of these test statistics. Analysis on simple examples where the truth is known demonstrates the potential gain in power obtained by including an extra dimension. Furthermore, it is found that a beneficial combination may be to combine two *χ*^2^ statistics, where the second one comes from the ability of a second model to describe data from the first, tested, model. This leads to a new and more general motivation for the log-likelihood ratio test (LHR), in the more general sense of non-nested nonlinear models. Importantly, our 2D approach allows for an easy illustration of when and why a combination of tests is advantageous, and when and why an additional model is helpful. Finally, our results and new approaches are also repeated and illustrated on a previously published example from insulin signaling, with real data, and with a biological question that now is resolved statistically for the first time.

## Methods

### Theoretical Setting

The herein presented bootstrap approach is applicable to any predictor-based model, *i*.*e*. for any model that can return a vector of predicted outputs $\hat{y}(\theta )$, given a vector of parameters *θ*. Importantly, this includes both linear and nonlinear, as well as static and dynamic, models. Nevertheless, since most models in systems biology are based on nonlinear ordinary differential equations (ODEs) [38], we here present the method in an ODE-based framework. In the results section, examples of both linear, nonlinear, static, and dynamic models are included.

Let the states in the model be denoted *x*, and let the time derivatives, $\dot{x}$, of these states be governed by a nonlinear smooth function, *f*. The states, *x*, usually correspond to the amounts or concentrations of particular molecular compounds. The function *f* is usually given by summing up kinetic rate expressions of the involved compounds, assuming mass action laws, or in some cases, more detailed rate equation expressions such as Michaelis-Menten dynamics [39]. Let the function *f*(*x*,*θ*,*u*) depend on the states, the parameters, and some input signals, *u*. Let the initial values for the states be denoted *x*_0_, and note that these most often are defined as part of the parameter vector *θ*. Finally, let the model outputs, $\hat{y}$, be determined by a smooth nonlinear function *g*(*x*,*θ*,*u*), which, just like *f*, may depend on the states, the parameters, and the inputs. With these notations, the state-space description of the model may be written as:

(1) $$\begin{array}{l} \;\overset{˙}{x}=f(x,\theta ,u) \end{array}$$

(2) $$\begin{array}{l} x(0)=x_{0} \end{array}$$

(3) y^=g(x,θ,u)

The noise, *v*, is assumed to enter only additively, and only in the measurement equations. Hence, with the measured output denoted as *y*, the assumption is that

(4) $$y(t)=\hat{y}(t,\theta )+\nu (t),\;\nu ∼D$$

for all *t*, and where *ν* follows the distribution *D*. A model, M(θ), is defined by the specific choice of the functions *f* and *g*, *i*.*e*. by the model structure , and some parameters, *θ*.

### Model implementation

All models have been implemented and analyzed using MATLAB®; R2011b [40]. Static models were fitted using standard linear regression methods, such as *polyfit*. ODE models were implemented using the Systems Biology Toolbox (SBTB) [41] and the add-on package SBAO. Parameters of ODE models were estimated using the global optimization algorithm *simannealingSBAO*, available in SBTB, by minimizing the *χ*^2^-test statistic.

### Bootstrap setting

A bootstrap sample, *b*, is an artificial vector of observations. A set of such bootstrap samples, $B_{i}$, is generated with the intent of representing the natural variation of the experimental data set, according to some specific procedures and assumptions. Here we consider parametric bootstrap samples, *i*.*e*. samples that have been generated from a specific model structure, denoted $M_{i}$, whose parameters have been fitted to agree with the experimental data. If nothing else is noted, the default set size, also called cloud size, used in this paper is 1000 samples per set.

The bootstrap samples are generated by adding noise, drawn from the assumed distribution *D*, to a simulated output of a given model. In this paper, the assumed noise distribution is Gaussian with a mean of 0, and a standard deviation of 0.5 and 0.75 for the static and dynamic case, respectively. These noise level were chosen to be in the order of 5-10% of the average model output. Conversely, the assumed noise level for the insulin signaling test case corresponds at each time point to the noise in the experimental data. However, for the first and second time point, where the signal has been normalized, and the noise therefore is zero, an average noise level for the data set is assumed.

Each bootstrap sample corresponds to a new realization of the noise with the same measurement signals and time points as in the observed data. The empirical distribution of any given test statistic, such as the *χ*^2^ or DW, is obtained by fitting the model of interest to all bootstrap samples, and then for each fit, calculating the appropriate test statistic.

### Empirical testings and conceptual basis behind the methods

**Figure 2:**
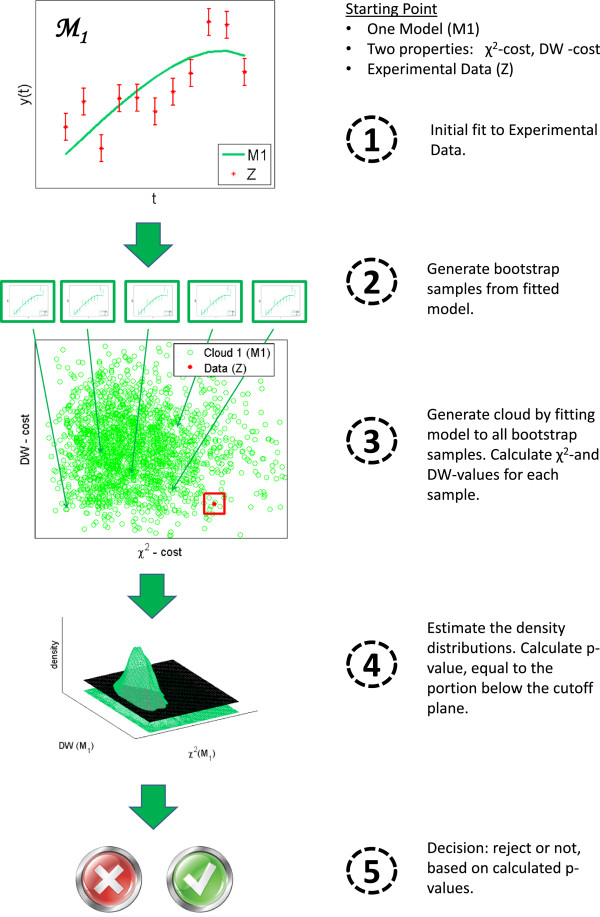
**A graphical summary of the proposed method steps for a 2D** ***χ***^**2**^ ***vs* DW analysis.** Encircled numbers correspond to the steps described in the model algorithm. The starting point is some data set, , a model structure, $M_{1}$, to be investigated, and two test statistics. First the model is fitted to the experimental data and then the fitted model is used to generate bootstrap samples. Next, the model is fitted to all bootstrap samples. The resulting two-dimensional cloud is used to generate a density estimation. The cutoff plane is defined as the equidensity contour that goes through the coordinates of the experimental data (red square). The corresponding p-value is equal to the portion of the distribution below the plane, *i*.*e*. outside the corresponding density contour line. The p-value is then used for decision on whether or not to reject either model.

A statistical test is a formal procedure for checking if a null hypothesis, here denoted $H_{0}$, can be rejected. In practice, a test maps a given set of observations, denoted , to a test statistic, T(Z). A p-value for a given test statistic is the cumulative probability of that value and all other values that are even more extreme, given the distribution under $H_{0}$, where $H_{0}$ typically corresponds to the hypothesis that the model you are testing is true. In a bootstrapping environment we construct these distributions empirically, as described in the section Bootstrap setting, rather than using analytical expressions for them (see also Figure 2 and the detailed descriptions below).

More specifically, if the null hypothesis that the model is true is correct, then bootstrap samples, generated from the fitted model, actually represent real samples from the true Data Generating Process (DGP). Thus, under $H_{0}$, the joint distribution of any number of Goodness Of Fit (GOF) statistics represents the distribution that we would expect to see if we were able to repeatedly sample from the true DGP, and each time were to fit our model and calculate a corresponding vector of GOF statistics. We can therefore calculate the probability, under $H_{0}$, of seeing a vector of GOF statistics at least as extreme as the original observed vector of GOF statistics, *i*.*e*. a p-value.

These p-values are calculated by estimating the densities of these GOF-distributions and then, as described for each test below, evaluate these at the coordinates of the observed data  to obtain the desired p-value. The null hypothesis is then rejected if the observed vector of test statistics is very unlikely under $H_{0}$. Usually this amounts to specifying a significance level *α* and checking whether the observed value(s) is more extreme than the corresponding threshold.

In this paper we consider a seemingly unexplored potential with bootstrap approaches: that they, unlike analytical approaches, allow for an easy calculation of the joint distribution of several test statistics. Consider a vector of *k* such test statistics, ( $T_{1},T_{2},\ldots T_{k}$). Given the null hypothesis that the tested model is true, one can then generate data that is assumed to come from the true DGP, and for each data series calculate corresponding values for all these *k* test statistics. These values then form vectors of values, and each vector constitute a point in a *k*-dimensional space. These points together form an approximation of the true *k*-dimensional distribution if the null hypothesis is true. Finally, the experimental data series  also corresponds to such a point, and we can use a multi-dimensional density estimation to see whether or not it is realistic to assume that the experimental point lies within the obtained distribution. This idea can in principle be used for any number of combined test statistics, but the computational cost of approximating a multi-dimensional distribution grows quickly with the dimension. Therefore, we here limit ourselves to combinations of two test statistics, *i*.*e*. to *k* = 2 and to 2D distributions.

### One-dimensional tests of a single model

#### The bootstrapped *χ*^2^-test

The *χ*^2^-test evaluates the size of the residuals, which are defined as the differences between the measured and predicted outputs:

(5) $$r_{i}(t_{j})≔y_{i}(t_{j})-{\hat{y}}_{i}(t_{j},\theta )$$

The test statistic, $T_{\chi^{2}}$, is given by the residual sum of squares

(6) $$T_{\chi^{2}}=\underset{i,j}{\sum }\;{\left(\frac{y_{i}(t_{j})-{\hat{y}}_{i}(t_{j},\theta )}{\sigma_{i}(t_{j})}\right)}^{2}$$

where the summation runs over all time points, *t*_*j*_, and all measurement signals, *y*_*i*_. An empirical distribution of $T_{\chi^{2}}$ is obtained by generating bootstrap samples from a model and fitting this model to the samples, as described above. The resulting distribution is evaluated using MATLAB and the empirical cumulative distribution function, *ecdf*. A p-value, $p_{\chi^{2}}$, under $H_{0}$, is obtained by considering a right-tailed (unless otherwise specified) distribution and interpolating the value of the cumulative empirical distribution at the coordinate corresponding to the *χ*^2^-value of the original data set.

#### The bootstrapped Durbin-Watson test

The DW test can be used to test whether the residuals in Equation (5) are too correlated or anti-correlated. The test statistic, $T_{\text{dw}}$, is given by

(7) $$T_{\text{dw}}=\frac{\sum_{i,j>2}{\left(r_{i}(t_{j})-r_{i}(t_{j-1})\right)}^{2}}{\sum_{i,j}r_{i}{\left(t_{j}\right)}^{2}},\;0\leq T_{\text{dw}}\leq 4$$

The numerator is a measure of the covariance of adjacent residuals, the denominator for the respective variance. For perfectly uncorrelated residuals the test statistic is equal to two. A value close to zero indicates a positive correlation, whereas a value close to four indicates a negative correlation. In this paper we have chosen to look only at correlation, and not at anti-correlation and therefore chosen a left-tailed distribution. An empirical distribution of $T_{\text{dw}}$ is obtained by generating bootstrap samples from a model and fitting this model to the samples, as described above. The resulting distribution is evaluated using MATLAB and the empirical cumulative distribution function, *ecdf*. A p-value, *p*_*dw*_, under $H_{0}$, is obtained by considering a left-tailed distribution and interpolating the value of the cumulative empirical distribution at the coordinate corresponding to the DW value of the original data set.

#### Simplistic combinations of bootstrapped tests

In this paper, p-values obtained from an empirical 1D *χ*^2^-distribution and an empirical 1D DW distribution are combined in various ways. Four of these ways are referred to as simplistic. These combination tests are defined as follows:

$$\begin{array}{l} \;p_{\text{min}}:=\text{min}\;\left(p_{\chi^{2}},p_{\text{dw}}\right)\\ \;p_{\text{max}}:=\text{max}\;\left(p_{\chi^{2}},p_{\text{dw}}\right)\\ p_{\text{mean}}:=\left(p_{\chi^{2}}+p_{\text{dw}}\right)/2\\ \;p_{\text{prod}}:=p_{\chi^{2}}*p_{\text{dw}} \end{array}$$

and the interpretations and motivations behind the combinations are described in the corresponding section in Results (Simplistic combinations of bootstrapped tests).

### Two-dimensional tests of a single model

#### Bootstrapped two-dimensional *χ*^2^ *vs* DW test

Now follows a description of the novel algorithm for a joint two-dimensional *χ*^2^ *vs* DW test. Although described as a combination of these two specific tests, the method is generalizable to any combination of two test statistics, by simply replacing one or more of the test statistics. Our proposed algorithm consists of the following steps (Figure 2). 

Algorithm:

 Given a model, $M_{1}$; an experimental data set, ; two test statistics, $T_{\chi^{2}}$ and $T_{\text{dw}}$; and a significance level *α*:

1. Fit the model to the original data set  and let ${\hat{\theta }}_{M_{1}}$ denote the estimated parameter vector. Calculate the statistics $T_{\chi^{2}}^{M_{1}}(Z)$ and $T_{\text{dw}}^{M_{1}}(Z)$ according to (6) and (7) respectively.

2. Use $M_{1}({\hat{\theta }}_{M_{1}})$ to generate a set of bootstrap samples. This set is denoted $B_{1}$.

3. Fit $M_{1}$ to each bootstrap sample, *b*, in $B_{1}$ and calculate the corresponding test statistics for each fit. This results in one set of *χ*^2^-values and one set of DW-values, which together form a two-dimensional cloud: 

 • $C_{1}$, cloud 1, consisting of $T_{\chi^{2}}^{M_{1}}(B_{1})$ and $T_{\text{dw}}^{M_{1}}(B_{1})$.

4. Estimate (see below) the two-dimensional empirical distribution $\rho_{1}(C_{1})$. Let $\rho_{1}(Z):=\rho_{1}(T_{\chi^{2}}^{M_{1}}(Z),T_{\text{dw}}^{M_{1}}(Z))$ denote the obtained density at the coordinate corresponding to the *χ*^2^ *vs* DW values of the original data set . For the given distribution, we define the cutoff plane as the equidensity contour that goes through $\rho_{1}(Z)$.

5. Using the two-dimensional distribution, calculate the p-value for the given model $M_{1}$,

(8) $$\begin{array}{l} p_{\chi^{2}-\text{dw}}^{M_{1}}:=\int_{\rho_{1}<\rho_{1}(Z)}\rho_{1}\left(T_{\chi^{2}}^{M_{1}}(B_{1}),T_{\text{dw}}^{M_{1}}(B_{1})\right)dT_{\chi^{2}}^{M_{1}}dT_{\text{dw}}^{M_{1}} \end{array}$$

If $p_{\chi^{2}-\text{dw}}^{M_{1}}<\alpha$, then $M_{1}$ should be rejected.

#### Two-dimensional density estimation

The two-dimensional density of a cloud is estimated continuously with a smooth Gaussian kernel [42,43], and evaluated over a grid, *i*.*e*. a 2D histogram. The integral in Equation 8 is then approximated by summing over all bins. The total volume is then normalized to one.

#### Two-dimensional p-value calculation

The calculations of p-values in 2D introduces some new considerations, and a few comments are in order. Consider Figure 1, and the property A, considered as a 1D distribution. Then, the probably most common way of calculating the p-value is *p* = 1 - *p*(*A* < *A*(*Z*)). Turning to 2D distributions, this formula can no longer be used, since there now are two properties, A and B. Instead a more general formula is needed. One such option is to use some formula based on the probability density function, *ρ*. Then the corresponding formula is *p* = 1 - *p*(*ρ* > *ρ*(*Z*)) = *p*(*ρ* < *ρ*(*Z*)) (Equation 8). In general, the p-value should give the probability that the obtained value, or an even more extreme one, is found, under the given null hypothesis, and this is ensured by both the 1D and 2D formulas. Note, however, that the 2D formula, using *ρ*, includes all regions of low density, even the ones where the model is surprisingly good, similar to a two-tailed test in one dimension. A more detailed discussion on these issues is found in the Discussion, and in Additional file 1: Figure S4.

### Tests involving two models

#### Bootstrapped two-dimensional *χ*^2^ *vs* *χ*^2^ test

Our proposed method for a two-dimensional *χ*^2^ *vs* *χ*^2^ test is similar to the two-dimensional *χ*^2^ *vs* DW test, where the DW test statistic has been replaced by the *χ*^2^-statistic of a second model. The detailed steps are explained in the Additional file 1 and in Additional file 1: Figure S1.

#### Bootstrapped log-likelihood ratio test

Given some data , and two models $M_{1}$ and $M_{2}$, an empirical distribution of the LHR, $T_{\text{LHR}}$, is obtained by generating bootstrap samples from either model ( $H_{0}$) and fitting both models to the samples, as described above. The resulting distribution of log-likelihoods (*χ*^2^-differences) are evaluated using MATLAB and the empirical cumulative distribution function, *ecdf*. A p-value, *p*_*LHR*_, under $H_{0}$, is obtained by considering a two-tailed distribution and interpolating the value of the cumulative empirical distribution at the coordinate corresponding to the LHR value of the original data set. These steps are explained in detail in the Additional file 1.

### Test cases

#### Static models

**Figure 3:**
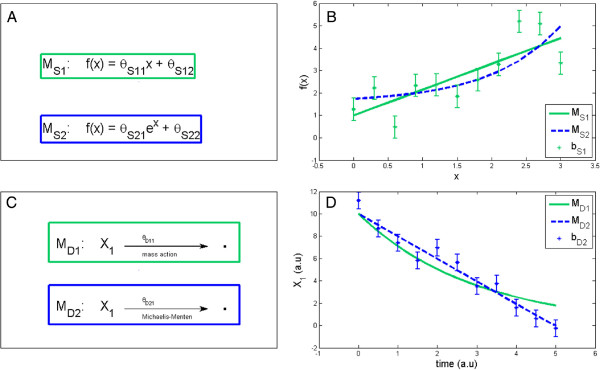
**The static and dynamic examples where the truth is known.** **(A)** The static model structures, where *x* and *f*(*x*) are the input and measured output, respectively, and where the *θ*s are unknown parameters to be estimated from the data. **(B)** Vertical lines are data points (mean plus/minus standard deviation) from an example of a bootstrap from the model structure $M_{S1}$, and the green and blue lines are corresponding fits from the two model structures. **(C)** The two dynamic model structures, involving a decay of the substrate *X*_1_, which occurs via mass action or Michaelis-Menten kinetics, respectively. **(D)** Vertical lines are data points (mean plus/minus standard deviation) from a bootstrap generated by $M_{D2}$, and the blue and green lines are corresponding model fits by the two model structures. Equations and details regarding fits etc. are found in Materials and Methods.

Two static models are considered; $M_{S1}$, a straight line, and $M_{S2}$, an exponential curve (Figure 3A-B). 

$\underline{M_{S1}}:\;f(x)=\theta_{S11}x+\theta_{S12}=\hat{y}$

$\underline{M_{S2}}:\;f(x)=\theta_{S21}e^{x}+\theta_{S22}=\hat{y}$

#### Dynamic models

Two dynamic non-nested examples are considered; $M_{D1}$, mass action kinetics, and $M_{D2}$, Michaelis-Menten kinetics with one free parameter (Figure 3C-D). 

$\underline{M_{D1}}:\;{\dot{x}}_{1}=-\theta_{D11}x_{1},x_{1}(0)=10,\hat{y}=x_{1}$

$\underline{M_{D2}}:\;{\dot{x}}_{1}=\frac{-\theta_{D21}x_{1}}{0.01+x_{1}},x_{1}(0)=10,\hat{y}=x_{1}$

### Analyses of methods

#### The receiver operator characteristic

The power of a statistical test is often determined by the relationship between the false positive rate (FPR) and the true positive rate (TPR) [44]. A false positive is the rejection of a true model, whereas a true positive is the rejection of a false model. The dependency of the TPR on the FPR is called a Receiver Operator Characteristic (ROC) curve. The more concave the curve, *i*.*e*. the larger the Area Under the Curve (AUC), the better the discrimination between true and false models. Here, ROC curves are constructed by considering a large number of artificially generated data sets, on which two hypotheses are tested, of which one is the true underlying model. The obtained p-values for each hypothesis and data set are calculated and for any given FPR (*i*.*e*. p-value) the TPR is obtained.

#### Type I error rate

**Figure 4:**
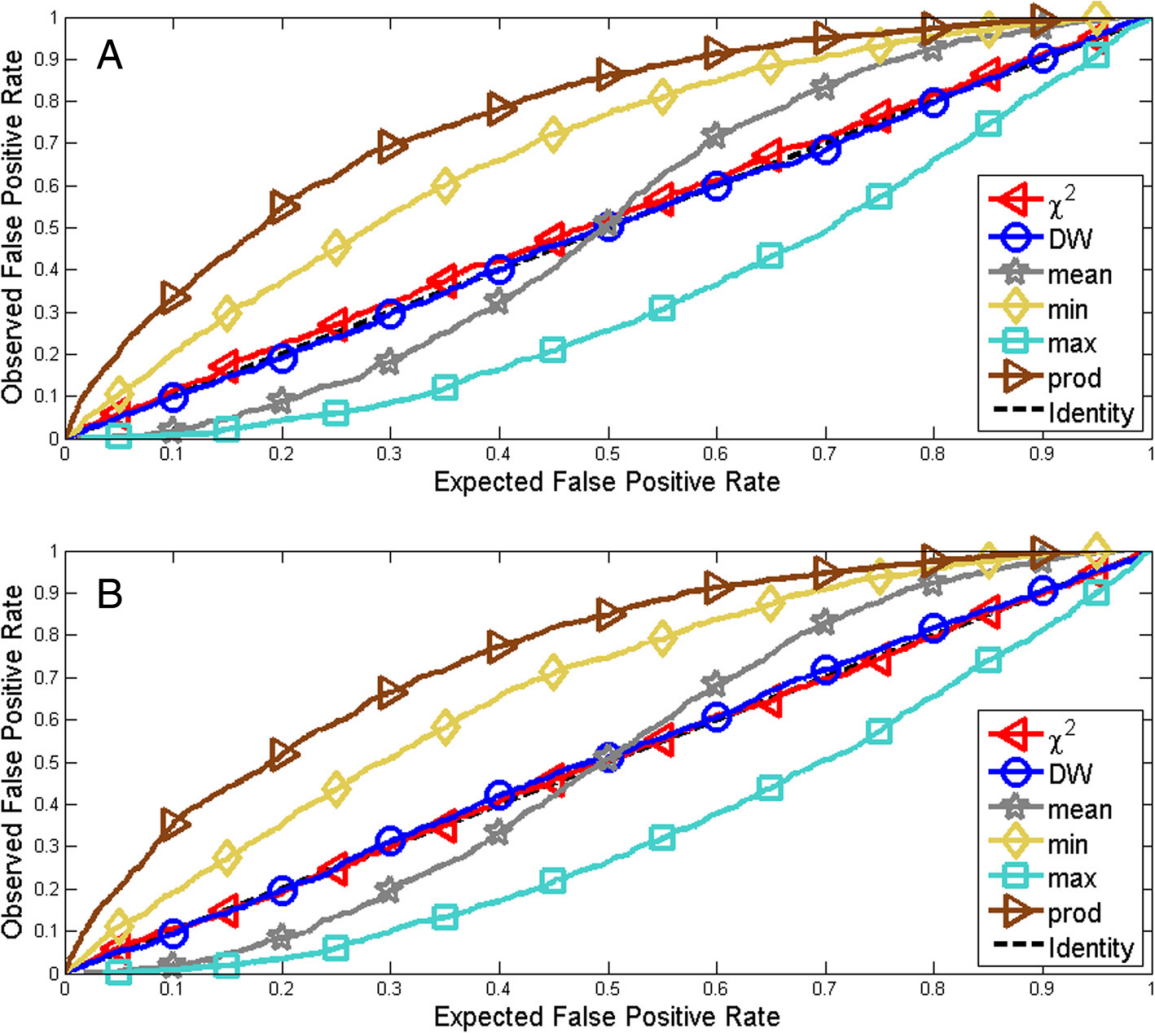
**Type I error rate plots for the single test statistics and their simplistic combinations.** Type I error plots show the Expected False Positive Rate (EFPR), which is the stated number of erroneous rejections of a true model, versus the observed number of rejections, Observed False Positive Rate (OFPR). **(A)** Static example **(B)** Dynamic examples. As can be seen, only the single test statistics, and none of the combinations, lie along the identity line. The simplistic combinations are therefore disregarded from further analysis.

For a given significance level *α*, it is expected that 100 ·*α* % of all true values would be rejected. If the observed FPR is higher than the expected FPR, the test is prone to making type I errors, and is considered liberal. In contrast, if the observed FPR is lower than the expected FPR, the test is considered conservative. This method property is evaluated by considering a large number of artificially generated data sets, where the true model is known, and where the calculated p-values thus can be compared to the underlying truth. Any given significance level, *i*.*e*. stated FPR, can thus be compared to the observed FPR, and the resulting relationship can be plotted in a graph (*e*.*g*. Figure 4). Ideally, the expected FPR should coincide with the observed FPR. A convex plot would indicate a conservative test, whereas a concave plot would indicate a liberal test.

## Results

### Test cases

In this paper we have employed an ensemble of various bootstrap methods on both static (Figure 3A-B) and dynamic (Figure 3C-D) test case models. We tested these approaches on static linear examples mainly for the following two reasons: firstly static models are common in science and our methods should therefore aim to be applicable to these kind of problems; secondly the solutions to the corresponding optimization problems are for these static linear examples unique and analytically attainable. In contrast, we also performed our analysis on two test cases in an ODE-framework. Parameters in ODE models usually have to be estimated, often by solving non-convex optimization problems, and it is then not guaranteed that the optimal solution will be found. In both settings, the number of parameters in the test case models were kept low, in order to more reliably evaluate the methods.

In each setting, static and dynamic, we let both models serve as the underlying truth to create 500 artificial data sets each. For each such data set both models served as $H_{0}$, and were consecutively fitted to the data, and the Goodness of Fit (GOF) was evaluated using various bootstrap approaches, starting with the simplistic combinations described earlier. Thus, for each setting and bootstrap approach, this resulted in 1000 true positives and 1000 true negatives, which were used to construct ROC curves to evaluate the power of the tests.

### Combining *χ*^2^ and DW statistics

The *χ*^2^-test is used to check whether the residuals, *i*.*e*. the differences between the model output and the data points, are too big. Conversely, the DW test is used the check if these residuals are too correlated. Both tests are useful in a model rejection scenario, and in the below analysis, looking at how these tests can be combined, the two tests are used as in their bootstrapped form (Methods).

#### Simplistic combinations are unsound

The first part of the analysis concerns the simplistic combinations: *p*_*min*_, *p*_*max*_, *p*_*mean*_, and *p*_*prod*_ (Methods). Although simple, these tests are not without interpretation, and several of them are what at first might seem like the obvious idea [34-37]. The options *min* and *max* corresponds to rejecting if either or if both individual tests reject, respectively. The *mean* could be thought of as a balancing between the two extremes, and *prod*, the product, could be thought of as the joint probability.

All these four simplistic combinations can be discarded based solely on an analysis of their observed and expected type I error rate. These rates are plotted for all four tests in Figure 4, and the interpretations of these curves is as follows. If the plotted lines lie away from the identity line, the expected false positive rate does not coincide with the observed false positive rate, and if this deviation from the identity line is large we call the test unsound. A large deviation means one of two things: either the test is liberal (if the line is above the identity line), or the test is conservative (if the line is below). A liberal method is generally regarded as unacceptable, since one wants to be able to trust rejections, but a small level of conservativeness could be accepted, so long as the test is useful. In both the static (Figure 4A) and the dynamic (Figure 4B) case, the tested combinations are unsound. The *min* (yellow diamonds) and *prod* (brown triangles) approaches are strikingly liberal, the *max* approach is highly conservative (cyan squares), and the *mean* (gray stars) switches from below to above. These plots should be compared to the single tests: *χ*^2^ (red triangles) and DW (blue circles) which lie along the identity line. This difference between the single tests and the simplistic combinations clearly illustrates that the deviations from the identity line are big. Since the results are essentially the same for both the static and dynamic cases, the results were deemed sufficiently convincing to be able to reject all of the tested simplistic approaches as unsound.

#### A two-dimensional approach is both sound and informative

**Figure 5:**
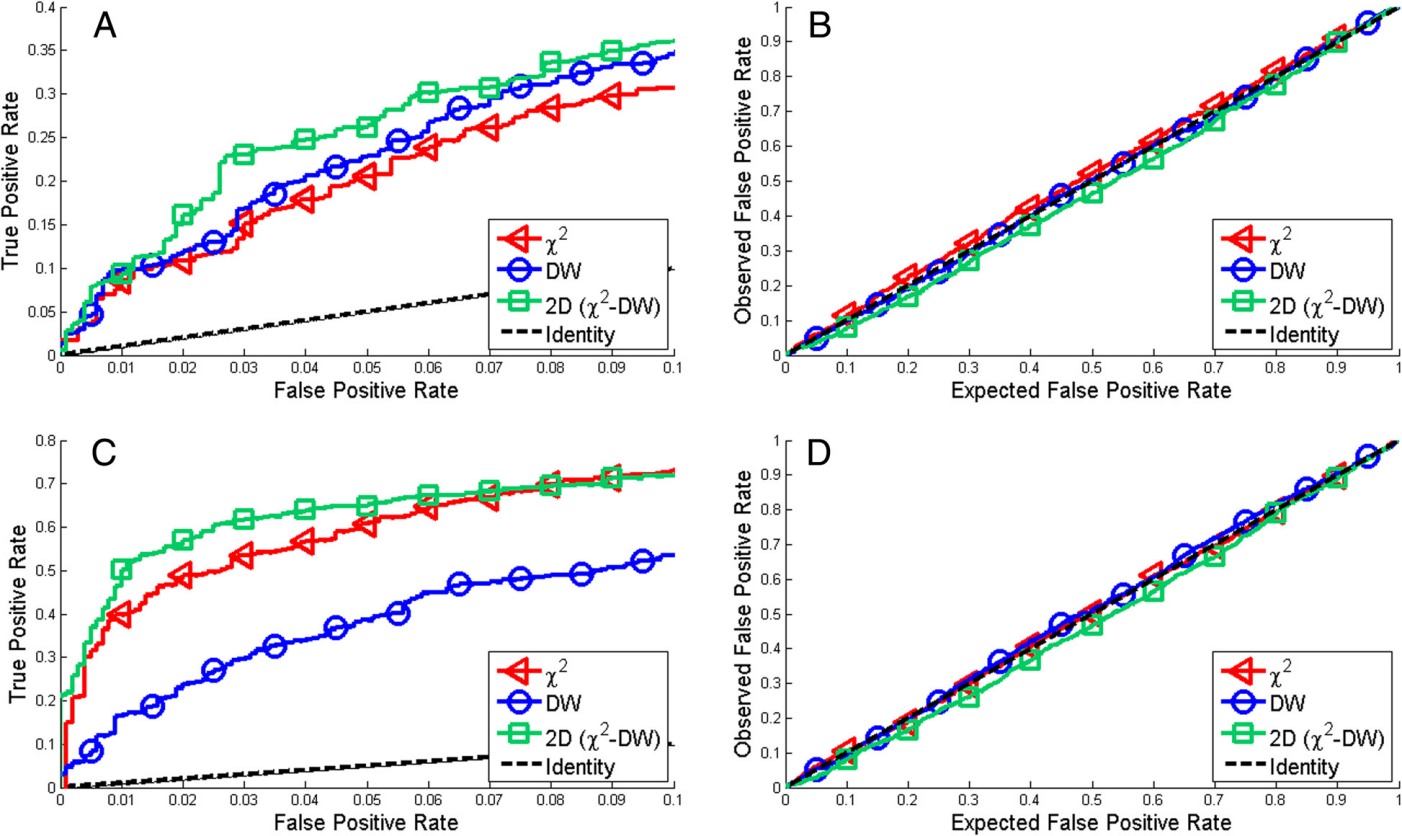
**ROC and Type I error rate curves for the 2D** ***χ***^**2**^ ***vs* DW analysis (green squares) compared to its two single constituent tests,** ***χ***^**2**^** (red triangles) and DW (blue circles).** The ROC curves **(A,C)** show the ability of the different tests to successfully reject the models that should be rejected (TPR), while rejecting as few as possible of the true models (FPR). In other words, a steep rise, and a high AUC is evidence of a good test. Since tests in practice only are used with p-values below 0.05, only the first part of the plot is shown (FPR <0.1). The Type I error rate plots **(B,D)** examine the same ability of producing correct p-values as in Figure 4. However, the combination used here (2D *χ*^2^ *vs* DW, green squares) does lie close to the identity line, together with the single test statistics. The upper plots, **(A,B)**, show the results for the static example, and **(C,D)** for the dynamic example. As can be seen, the 2D *χ*^2^ *vs* DW test is consistent **(B,D)** and superior to both of its constituents tests, which is evident from the greater AUC in both the static and the dynamic case **(A,C)**.

The second part of the analysis considers the 2D analysis, combining the *χ*^2^ and the DW tests (Methods, Figure 2). Although a precursor to the 2D methods presented herein has been mentioned and outlined in previous papers [2], this is the first time all the implementation details have been solved, and the performance of the method is tried on examples. One of the novel details concerns the density estimation. In contrast to the simplistic combinations, this 2D approach is sound, or only slightly conservative, for both the static (Figure 5B) and the dynamic (Figure 5D) case. The conservativeness is tolerated, since the test is informative, as can be gathered from the Receiver Operator Characteristic (ROC) curves in Figure 5A and C. These ROC curves are to be interpreted as follows. On the x-axis, the rate of erroneous rejections are plotted; this value is therefore to be as small as possible. On the y-axis, the rate of correct rejections are plotted; this value is therefore to be as high as possible. Taken together, this means that the AUC should be as big as possible, especially for the region of interest where the FPR is below 0.1. This region of interest was chosen because in biology *α* is rarely higher than 0.05. From Figure 5A,C it is clear that the new 2D approach (green squares) outperforms both *χ*^2^ (red triangles) and DW (blue circles) considered as individual tests.

### Introducing a second model

#### Replacing the DW test statistic with the *χ*^2^-test statistic of a second model

The above 2D approach (Figure 2) can be generalized to combinations of other tests as well. An important aspect of this is that one can consider a combination of two *χ*^2^ tests, where the second *χ*^2^ test comes from a second model, $M_{2}$ (Methods, Additional file 1 Methods, and Additional file 1: Figure S1). It is therefore intuitively sensible to test whether such a usage of two models is an advantageous usage of this 2D approach. This property of one model’s ability to imitate the behavior of a second model is known as model mimicry, and the idea of using this in a model *selection* setting has been utilized by *e*.*g*. [11].

This second model can in itself be an uninteresting model, *i*.*e*. we are then not necessarily interested in the second model as an explanation of the original data, but only in how it interacts with the model being tested. Such a model is called a *help model*. Alternatively, the second model could be a competing model and its ability to describe the original data is then of equal importance as that of the first model. If this latter situation is the case, one would typically perform the analysis with both models serving as $H_{0}$, generate bootstrap samples from each model, and so on (Additional file 1: Figure S1). This version of the 2D test then becomes a form of model comparison, even though there are important differences. For instance, this 2D analysis, unlike model discrimination tests like the conventional non-bootstrapped LHR, can result in all four cases of rejecting none, either, or both of the models. In contrast, a conventional non-bootstrapped LHR can only result in the rejection of the simpler of the two models, or none of them. Furthermore, in this new setting, the two models do not have to be nested, *i*.*e*. one of the models does not have to be a special case of the other, and the models can be general nonlinear ODEs.

**Figure 6:**
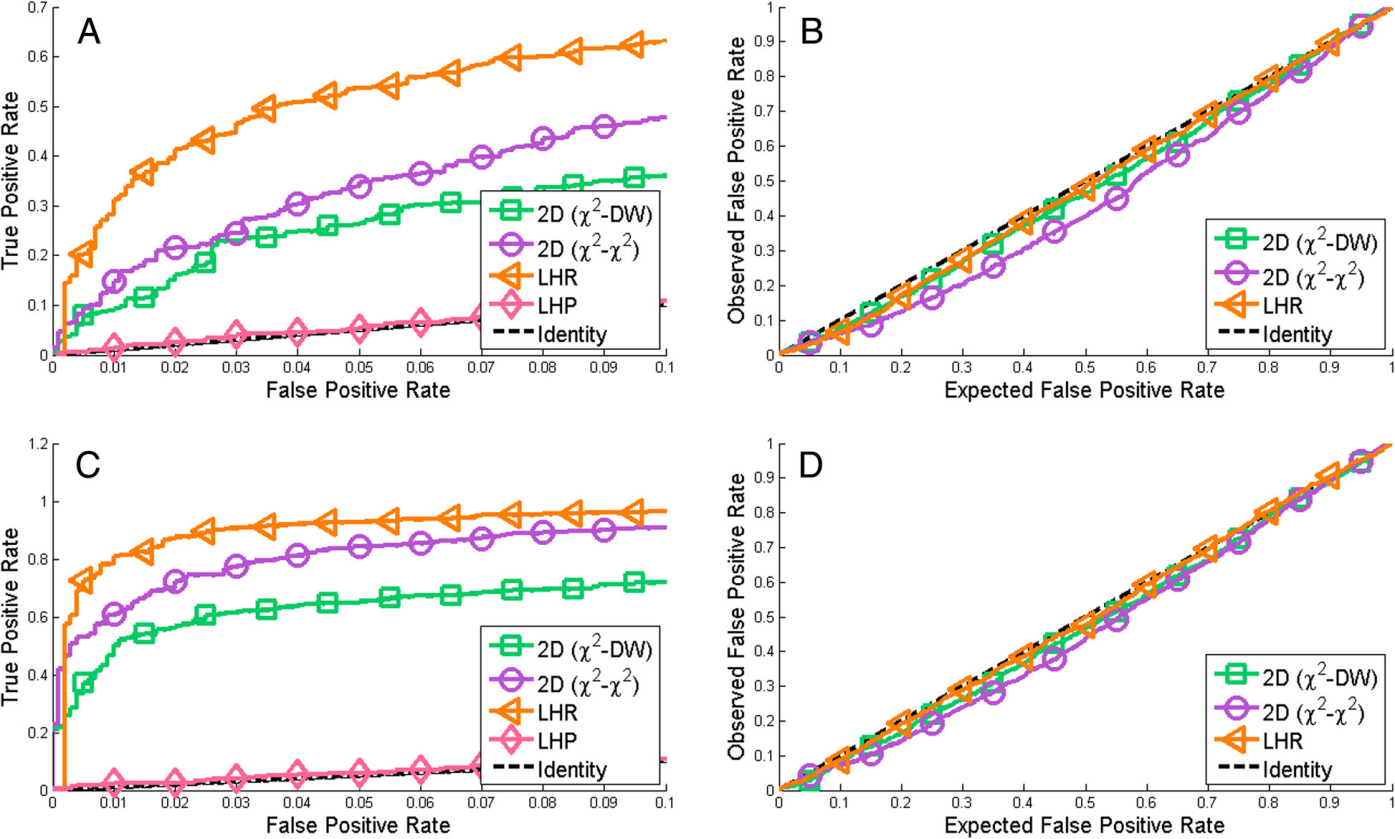
**ROC and Type I error rate curves for the approaches utilizing a help model compared to the 2D** ***χ***^**2**^ ***vs* DW test.** The structure and interpretation of the plots are the same as for Figure 5: **(A,C)** are ROC curves, **(B,D)** are type I error curves, **(A,B)** are for the static, and **(C,D)** for the dynamic example. As can be seen, all methods are consistent, or weakly conservative. The ROC curves show that the 2D *χ*^2^ *vs* *χ*^2^ test (purple circles) is better than the 2D *χ*^2^ *vs* DW test (green squares), and that the bootstrapped LHR (orange triangles), is best of all. In contrast LHP (pink diamonds) is completely uninformative, as it lies along the identity line.

The results on the same dynamic and static examples as before are plotted in Figure 6. As can be seen from Figure 6B and D, this 2D *χ*^2^ *vs* *χ*^2^ method (purple circles) also has some slight issues with conservativeness, but from Figure 6A and C, it is clear that this slight conservativeness should be tolerated: the new 2D version outperforms the previous 2D method (the purple circles lies above the green squares, and thus has greater AUC).

#### The bootstrapped LHR test is the best approach in the case of a good help model

The final test included in this comparison is a bootstrapped version of the LHR (Methods, Additional file 1 Methods). This method has no issues with conservativeness (Figure 6B and D, orange triangles), and outperforms all the other methods in terms of a ROC analysis (Figure 6A and C, orange triangles are on top).

#### New motivation for the LHR test in the more general case of bootstrapping non-nested models

**Figure 7:**
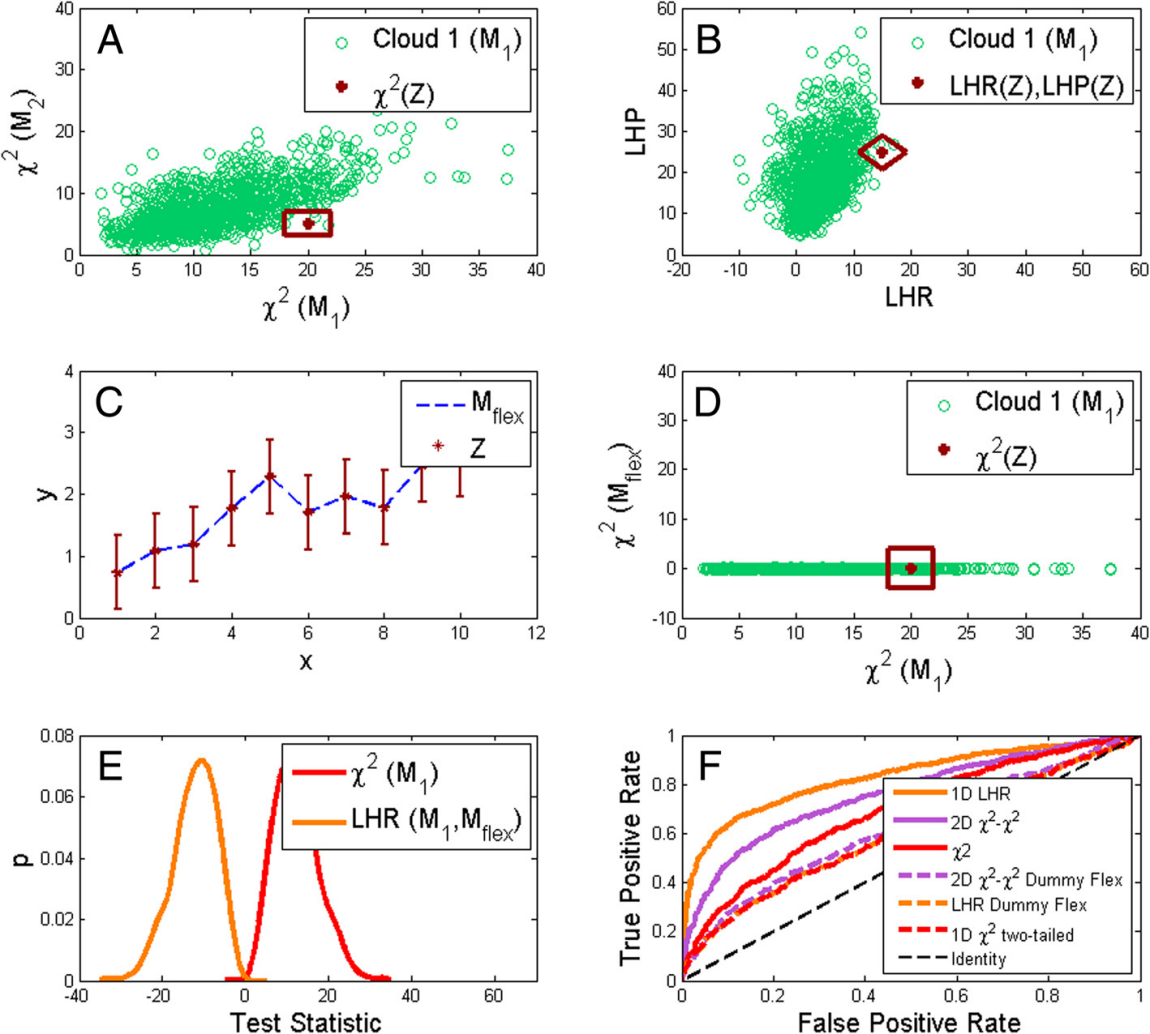
**Choice of help models.** **(A,B)** A beneficial help model. Green circles correspond to bootstrap samples from a static example cloud. The red dot correspond to a measured data point example, that makes use of the tilting of the green cloud away from the axes. The shape of the green cloud and the distance to the red symbol is invariant when one transforms from the *χ*^2^ *vs* *χ*^2^ plane **(A)** to the LHR *vs* LHP plane **(B)**. Importantly, the distance between the red symbol and the green cloud can be seen in the 1D projection to the LHR plane. **(C-F)** Illustration of how a bad, hyper-flexible, help-mode can be understood. **(C)** Model fit (blue dashed) to data (red vertical lines) for the hyper-flexible help-model. **(D)** same as in **(A)** but where the help-model is the hyper-flexible model. This cloud does not lie away from the axis, but parallel to the x-axis. Hence, all information is already contained within one dimension, and transforming to the LHR *vs* LHP plane will not help. **(E)** The 1D *χ*^2^ test (red) and the LHR (orange) empirical distributions for the case of a hyper-flexible model, each being the mirror image of the other. **(F)** A ROC analysis comparing a good help model with the bad hyper-flexible help-model in the static example. As before, 1D LHR (solid orange) is on top, above the 2D *χ*^2^ *vs* *χ*^2^ plot (solid purple) and the 1D *χ*^2^ (solid red). Those are the plots with the good help-model. The new plots with the bad hyper-flexible help-model lie below, and LHR becomes equally bad as the two-tailed *χ*^2^ test (the orange dashed and red dashed lines are superimposed). The 2D *χ*^2^ *vs* *χ*^2^ test (dashed purple) is slightly better, but still worse than the *χ*^2^ test.

It is now clear that there are examples where the LHR is advantageous to the 2D *χ*^2^ *vs* *χ*^2^ analysis; let us now understand why this is the case. At a first look, it seems like it should be the other way around: that the 2D *χ*^2^ *vs* *χ*^2^ analysis should be superior, almost by definition. The reason for this is that the information in the LHR is contained in the 2D *χ*^2^ *vs* *χ*^2^ analysis. The close relationship between the two methods can be seen by comparing a cloud from the analysis plotted in the *χ*^2^ *vs* *χ*^2^ plane (Figure 7A), with the same cloud plotted in the LHR *vs* log-Likelihood Product (LHP) plane (Figure 7B). As can be seen, the shape of the cloud and its relation to the red dot is identical, only tilted 45 degrees. This relation also follows from simple linear algebra.

A further inspection of the cloud in Figure 7A, which is taken from the static example, reveals that the cloud is of a character that makes a 2D approach superior to the individual tests: just as the cloud in Figure 1, the cloud in Figure 7A lies away from the axes, and the red dot can be distinguished better in a 2D analysis, than by looking along the individual axes. However, when the cloud has been tilted to the LHR *vs* LHP plane (Figure 7B), the red dot can be separated from the cloud when considering only one of the directions: the LHR direction. That this preservation of the information is preserved when projecting the 2D cloud to the LHR line is also corroborated by the fact that the LHP is, for this example, completely non-informative (the pink diamond lines in Figure 6A,C follow the identity line). In other words, the 1D LHR has extracted virtually all the relevant information of the 2D cloud.

All this means that if there would not be any price to pay for doing a 2D analysis, the LHR and 2D *χ*^2^ *vs* *χ*^2^ analysis would be equally good. However, there *is* a price to pay for moving to a 2D bootstrapping analysis, and this is the reason why the LHR bootstrapping analysis is superior. There are several components to this price. First, the estimation of a distribution is more cumbersome. Second, and more importantly, this 2D estimation converges slower than a corresponding 1D density estimation. This has to do with the curse of dimensionality, which simply means that the same number of points quickly become more scarcely packed as the dimensions increase, and that a corresponding density estimation will be based on fewer and fewer neighboring points. This reduction in convergence speed can also be seen in Additional file 1: Figure S3, where the LHR has converged already with cloud sizes of ∼ 1000 data points, but where the 2D *χ*^2^ *vs* *χ*^2^ analysis requires at least two more orders of magnitude for its convergence. Finally, there are also other prices of moving to a 2D analysis, such as the inability to clearly define a one-sided or two-sided test (see Discussion). The existence of such additional factors is also reflected by the fact that the 2D test does not converge to the same ROC curve as the LHR test (Additional file 1: Figure S3).

#### Choosing the second model

Having established that the inclusion of a second help model may improve the statistical power of tests evaluating the *first* model, leads to the natural question of whether all help models would do. The answer to this is “no”: if the help model is too simple or too flexible, the advantage is lost, and the resulting model comparison tests - LHR or the 2D *χ*^2^ *vs* *χ*^2^ analysis - perform worse than the other sound tests presented herein.

As an example of this, consider the completely flexible model, which simply goes through all data points (Figure 7C). If this model, $M_{\text{flex}}$, is used as the help model instead of the suggested competing model in the static example, the 2D cloud collapses to a line: since the cost of the help model is always zero (Figure 7D). Therefore, there is no 2D advantage to make use of, and the LHR distribution will simply be zero minus the *χ*^2^ distribution (Figure 7E), and LHR thus performs as bad as the two-tailed *χ*^2^ test (Figure 7F, orange dashed line and red dashed line are superimposed).

In the Additional file 1, another simple help model is considered: a constant model that simply approximates a data-series with its mean value. Here, the picture is a little bit more mixed. For the static example, the model is too simple, and the two-model tests are in-advantageous (Additional file 1: Figure S5). For the dynamic test case, the constant model does provide some additional information: the 2D *χ*^2^ *vs* *χ*^2^ analysis performs slightly better, and the LHR test slightly worse, than the single *χ*^2^-test (Additional file 1: Figure S2A).

Finally, for all of the above examples with too simple or too flexible models, the 2D *χ*^2^ *vs* *χ*^2^ analysis is superior to the LHR test, showing that the LHR is more sensitive to the situation of having chosen a bad help model.

### Application to insulin signaling

**Figure 8:**
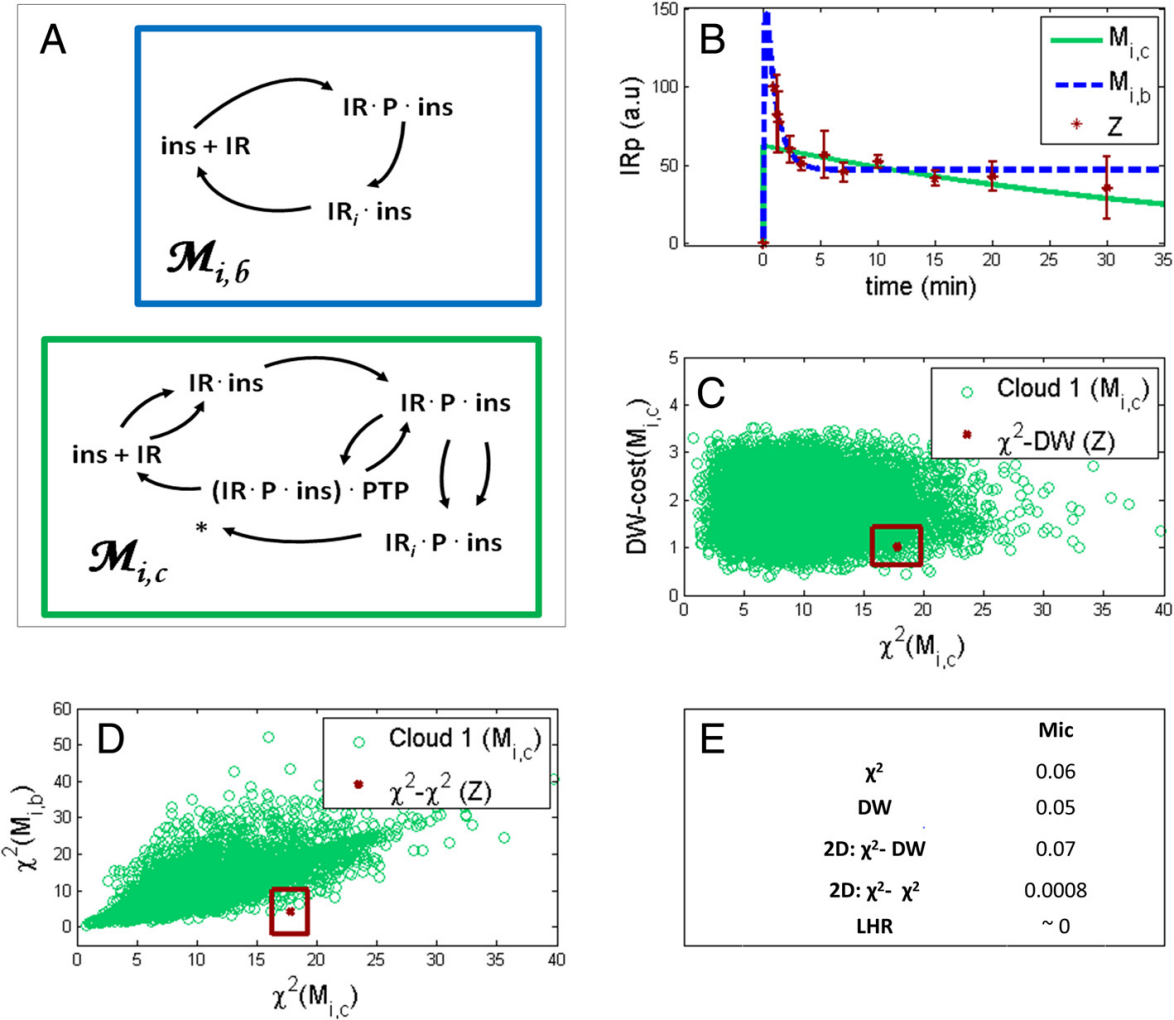
**Insulin signaling case.** Panel **(A)** shows the model structures of two models of early insulin receptor signaling, $M_{i,c}$ and the chosen help model $M_{i,b}$. This example was analyzed previously [45] and is of interest, since the rejection of $M_{i,c}$ would suggest that the recycling is a necessary mechanism to explain the data. Depicted in panel **(B)** is the experimental data Z (red error bars), and fits of $M_{i,b}$ (blue, dashed line) and $M_{i,c}$ (green, solid line). The measured data represent the increased auto-phosphorylation of the insulin receptor in response to 100 nM insulin in isolated primary human adipocytes, as measured by SDS-PAGE and immunoblotting. Panel **(C)** shows the bootstrapping cloud in the *χ*^2^ *vs* DW plane, when the bootstraps have been generated by $M_{i,c}$ (green circles, cloud size = 10^4^). As can be seen, the cloud lies along the axes, and there is no benefit of using a 2D analysis. Panel **(D)** shows the *χ*^2^ *vs* *χ*^2^ scatter plot of the $M_{i,c}$ cloud (green circles, cloud size = 10^4^) generated after fitting both models to bootstrap sets from $M_{i,c}$. The corresponding *χ*^2^-values for the experimental data (Z) is also plotted (red box). As can be seen, the cloud lies away from the axis, and the experimental data point explores the obtained direction. Panel **(E)** summarizes the results. As the clouds have indicated, the *χ*^2^ *vs* DW combination does not improve upon the individual tests, but still lies on the border of rejection. The *χ*^2^ *vs* *χ*^2^ tests on the other hand perform better than the individual tests, and the LHR is best of all.

As a real modeling example, we used data and models from a previous work [45]. In that paper we analyzed experimental data from insulin signaling in primary human adipocytes. Some of the experimental data are shown in Figure 8B. The data consist of a time series which displays an overshoot: the response increases rapidly from zero, and reaches a maximal value around 1 min, and then decreases to an intermediate steady state value. The observed response is caused by the addition of 100 nM insulin at time zero to previously unstimulated fat cells, and the measurements are performed using SDS-PAGE and immunoblotting to determine the degree of auto-phosphorylation of the insulin receptor (IR). The data are normalized such that the first point is zero, and the maximal value is 100. For further details regarding the data, we refer to [45]. Using mathematical modeling, we were able to reject several model structures aspiring to explain these data, and we used model selection tools, such as the Akaike Information Criterion (AIC) [46,47], on surviving competing hypotheses.

Here, we re-examined one of the models, $M_{i,c}$, from [45], that could previously not be unambiguously rejected. The model structure of $M_{i,c}$ and the chosen help model, $M_{i,b}$, are included also in this paper for convenience (Figure 8A). $M_{i,b}$, the smaller model, contains only three reactions: insulin binding with auto-phosphorylation of the receptor, internalization with dephosphorylation, and recycling back to the plasma membrane. $M_{i,b}$ fits to the data with a distinct overshoot, reaching an intermediate quasi-steady state after a couple of minutes (Figure 8B, blue dashed line). $M_{i,c}$, on the other hand, is considerably more detailed in terms of the binding, auto-phosphorylation, and internalization, but it does not contain any recycling. $M_{i,c}$ fits to the data in a qualitatively very different way (Figure 8B, green solid line). $M_{i,c}$ has its maximum almost immediately after stimulation, but thereafter never reaches any quasi-steady state as $M_{i,b}$ does. Instead the signal declines almost linearly during the observed time period. This example is of biological interest, since the rejection of $M_{i,c}$ would suggest that the recycling is a necessary mechanism to explain the data. This conclusion would follow because $M_{i,c}$ is a more complex, and a more realistic interpretation of the underlying biological system in all other aspects except recycling. In [45], an AIC analysis and a *χ*^2^-square test were performed on the various competing models and although $M_{i,c}$ was discarded based on its lack of agreement with data and the fact that inclusion of a recycling reaction yielded a better model, neither of these results were statistically convincing. Indeed, although the results pointed towards the rejection of $M_{i,c}$, it was pointed out already in [45] that a more accurate analysis would require a more generally applicable method such as bootstrapping.

In the re-analysis of this insulin signaling example, the analysis was done using all bootstrapped tests that have been found sound on the simpler test cases, where the truth was known. Unlike in those examples, here the truth is not known, but subsequent experimental analysis of the system has revealed that $M_{i,c}$ indeed should be rejected. The results are summarized in Figure 8E. As can be seen, a bootstrapped *χ*^2^-test yielded p = 0.06, and a bootstrapped (left-sided) DW test yielded p = 0.05, both on the border of rejection. A 2D *χ*^2^ *vs* DW analysis did in this case not improve this value because the resulting cloud and data point (Figure 8C) did not lie in an informative configuration (as explained in Figure 1), p = 0.065. Conversely, for the 2D *χ*^2^ *vs* *χ*^2^ the cloud and data point *did* lie in an informative configuration (Figure 8D). As a result, the two best methods on the test cases, the 2D *χ*^2^ *vs* *χ*^2^ and the bootstrapped LHR showed improved performance as compared to the above tests, yielding p-values that were clearly below the threshold (Figure 8E), p = 8∗10^-4^ and p ∼ 0 respectively. Also, the internal order of these methods was preserved: LHR was better than the 2D *χ*^2^ *vs* *χ*^2^. These findings are the same as in all tested examples with a sufficiently good help model, and speaks for their generality.

## Discussion

In this paper we have extended the traditional parametric bootstrap approach to also look at combinations of different test statistics, here exemplified by the *χ*^2^-test statistic and the DW test statistic. We have shown how simplistic combinations, as considered in the literature, of these two statistics are unsound, but that a two-dimensional empirical distribution, as enabled by the bootstrap setting, is both sound and informative. We have further shown that it is even more informative to replace the DW statistic with the *χ*^2^-statistic from a second model, resulting in a two-dimensional *χ*^2^ *vs* *χ*^2^ test. However, the one-dimensional bootstrapped LHR is found to be even better, and an analysis of this has led to a new motivation and understanding of the LHR, in the more general case of nonlinear and non-nested models.

A 2D bootstrap approach may be superior to a corresponding 1D approach, but there is in fact a trade-off between negative and positive aspects. One positive aspect is of course that a 2D analysis contains more information than a 1D analysis, since *e*.*g*. the insights contained in two statistics can be combined. Second, even though one in principle can combine test statistics in 1D as well, Figure 4 shows that the simplistic combinations give inconsistent results, and therefore are unacceptable. Conversely, a 2D-combination of the same test statistics is sound (Figure 5B,D). A third positive aspect of 2D combinations is that they have a higher power than either of the 1D tests including only one of the test statistics (Figure 5A,C). All these positive aspects are due to the benefit illustrated in Figure 1, where it is clear that only the 2D combination of the test statistics reveal that the indicated point lies outside the empirical distributions. There are, however, also several negative complications inherent in the addition of an extra dimension, and these complications are solvable to different extents. The first complication concerns the more complicated density estimation that is required in 2D, but this has been resolved in the herein presented 2D approaches. The second complication stems from the relative scarcity of neighboring points in two dimensions and its effect on the density approximation. If the same number of points is used to estimate a 1D distribution and a corresponding 2D distribution, the end result will be a worse approximation for the 2D distribution, simply because of the curse of dimensionality. This second complication can probably be completely overcome by adding more points to the estimation of the empirical distribution, but the addition of more points comes at the price of a higher computational cost. The third complication is that a right-tailed, left-tailed, or two-tailed tolerance interval cannot be clearly defined in two dimensions. This issue is described more below. Finally, the positive aspects of the additional dimension only appears if the resulting cloud does not lie along one of the axis, but as in Figure 1, in a direction that is not parallel to the axis. All in all, this means that the advantages of a switch to a 2D approach are not guaranteed, while the negative consequences *are* guaranteed. For this reason the choice between 1D and 2D is a trade-off.

Similarly to the issue of the second dimension, our results seem to indicate that the addition of a second model provides an advantage, compared to analyzing a single model based only on its own residuals, but there is in fact also here a trade-off between positive and negative aspects. The positive aspects are clear from the examples where we use a help model that is roughly equally good as the tested model: then the ROC curves have a higher area under the curve (Figure 6A,C), and because the model that should be rejected in the insulin signaling example gives a lower p-value (Figure 8E). These positive sides mean that the additional information provided by a second model provides valuable knowledge regarding the quality of the first model. More specifically, this arguably means that the second dimension from the second models in the examples implies 2D clouds that do not lie along any of the axes, but, as in Figure 1, in a direction that is not parallel to the axes. A final positive aspect of this bootstrapped two-model approach is that it goes beyond the capacity of a normal model comparison test, *e*.*g*. LHR, since the resulting conclusion can be that none, either, or both models are rejected. A standard model comparison test can never reject both models. The negative aspects of adding a second model are of two types: i) those that have to do with a 2D approach, and which are described above, and ii) those that are associated to the fact that not all additional models provide an improvement. As is clear from *e*.*g*. Figure 7F, it is clear that a poorly chosen model yields a worse test compared to a mere *χ*^2^-test. The second negative aspect is that it is for the non-nested cases not possible to know in advance when a model is good or poor help model. Here it should be re-stated that the 2D *χ*^2^ *vs* *χ*^2^ test is more robust towards bad help models than the LHR test in all examples tested herein. In summary, a help model should not be too flexible or too inflexible, and one can see whether the right complexity of the help model has been struck from the shape of the cloud: if it lies along one of the axes it is too flexible or too inflexible.

One of the negative aspects mentioned above needs a little further clarification: the definition of the empirical tolerance intervals, which are used to calculate the empirical p-values. First, the 1D versions of the methods that we are using either operate in a one-sided way (*χ*^2^ and DW, Additional file 1: Figure S4A), or a in a two-sided way (LHR, Additional file 1: Figure S4B). There is no obvious translation of sides and tails in a 1D distribution, to a 2D equivalent. We here adopt the definition of the 2D tolerance region(s) as the region(s) with highest probability density (Equation 8). In practice this is similar to a two-sided cutoff since one may reject a model because it is unrealistically good at describing the data, compared to the given noise level. However, there are differences, such as the possibility to have several fragmented regions instead of a single joint one. Therefore, when comparing our method with a 1D-version of the same test, one could consider defining the 1D tolerance interval(s) in a likewise manner (Additional file 1: Figure S4C-D), since this more closely mimics the cut-off we do in 2D. However, all comparisons here are done with the one-sided or two-sided 1D-versions of the methods, since it is those that are used in practice, and those that our method should out-compete.

A key validation step, and a demonstration of the usefulness of the results herein, is that they are corroborated on a real-world example, which now has been resolved statistically for the first time: we can now reject $M_{i,c}$ with a p-value that lies clearly below the threshold. We have confidence that this rejection of $M_{i,c}$ is correct, because in more recent works we have experimentally shown, by blocking internalization and measuring a downstream component, that recycling of the receptor does play a major role in insulin signaling [3]. We have also measured the amount of internalized insulin receptor and shown that there is far too little internalized IR to explain the observed overshoot. However, even though we in light of these new data have rejected this model, it is interesting to observe that it was possible, with our new method, to reject $M_{i,c}$ based only on the data available at the time of [45].

There are some limitations when interpreting the results that should be mentioned. First, the results are only shown for three example cases, and there is no guarantee that they hold for all other examples. Nevertheless, the results are essentially the same for all these three examples: 2D is better than 1D for the static and dynamic examples, and for all examples the tests with a non-extreme help model are better than the single rejection tests, and LHR is best of all. Therefore, since the examples include static, dynamic, linear, nonlinear, and real-world aspects, these overall trends probably have some generality. Second, the generality is also limited by the fact that we do not have analytical proofs for the results. This, however, is normal for bootstrap approaches. Third, another limitation with this approach is that it only considers the usage of a single help model or help statistic. However, this is not a major limitation, since we anyway only advocate the usage of these methods in special cases, where power and accuracy, rather than computational speed, are desired. In other words, our suggestion is to use this approach only in cases where you have a specific model that requires a more detailed analysis. Also, it is unlikely that a generalization of this approach to 3D would be beneficial, since then the price of estimating density in a high-dimensional space, and the corresponding slower convergence due to the curse of dimensionality, would be even more severe.

It is important to put our results in relation to the existing literature in related fields, such as statistics [17,19,47,48], systems biology [1,2,38], econometrics [15,49], mathematical psychology [11], phylogenetics [30,31,50] etc. First, our method is advantageous only in cases where you have a particularly important and challenging rejection case, where computational time is not a big issue. This stands in contrast to the typical situation of AIC and Bayesian Information Criterion (BIC), where a big number of models can be sorted through a simple criterion [44,46,47,51]. Similarly, the herein presented methods are not suitable to the sub-field of optimal experimental design for the purpose of improved model rejection, since such studies requires an optimization over different experimental designs, which in turn mandates less computationally heavy approaches [12,13,52]. Second, the perhaps most commonly used method for model rejection, the *χ*^2^-test, has a problem - that the degrees of freedom in the *χ*^2^ distribution usually is unknown [2] - but this problem is overcome by using the methods considered herein. However, this is not a new result, but is true for all bootstrap approaches, characterizing the distribution empirically. Third, there are a number of commonly used test statistics that we have not considered [18,24,53]. For instance, as an alternative to the DW test to measure correlation among the residuals, the whiteness and the run test may be used. It is still unknown whether our results for how to combine test statistics holds also for these other tests. The final, and now following, two comparisons with literature have to do with the LHR and with Bayesian approaches.

The herein presented analysis presents a new way of showing why and when the LHR is advantageous compared to an individual *χ*^2^-test, for the more general setting of nonlinear and non-nested models. Since LHR has been both extensively used and studied in the literature, it is important to relate this claim to previous results. LHR was first advocated by Neyman and Pearson in 1928 [15,48], and the basic *χ*^2^ distribution relation for nested linear models was known already in the 30’s [15,54]. These results were generalized to the important case of nonnested models by Cox in [16,17] and to the case of neither of the competing models being true by Vuong [15]. However, these results are of limited use, since they rely on analytical derivations of mean and variance terms [49], and the results by Vuong do not even apply to time-series models [15]. Also note that there are important cases where the traditional likelihood ratio test is not applicable, *e*.*g*. for usage in stochastic models based on the chemical master equation. All of these limitations can be avoided, by adopting a bootstrap approach. This approach basically only relies on the ability to draw bootstrap samples in a way that approximates the true data gathering process. The simulation based type of bootstrap approaches studied herein was originally proposed by Williams *et al.*[25]. The Williams approach has all the essential qualities of how we implement the bootstrapped LHR herein: both models are fitted to the data, and the fitted parameters are used to generate bootstrap samples that explicitly incorporates the null hypothesis that the used model is true, and finally both models are fitted to all bootstraps and corresponding distributions of LHR values are calculated. This approach has also been widely used using minor modifications [9,10,55], including an approach where the bootstrap samples are generated using drawing with replacement of the residuals [33]. There are also some papers where theoretical properties of the Williams approach have been investigated. For instance [49], shows that the empirical distribution of Williams asymptotically converges to the correct distribution under certain conditions. However, none of those papers use a 2D approach such as ours to achieve an intuitive understanding for why the LHR may be advantageous: that it incorporates the potential added value of the 2D approach compared to the individual *χ*^2^-tests, without paying the price of a 2D density estimation. The most important and novel part herein is perhaps that it allows the user to quickly check whether and why the bootstrapped LHR is advantageous or disadvantageous to use compared to the individual *χ*^2^-test: it depends on whether the second *χ*^2^-test yields a cloud that lies away from being parallel to the axes, which in turn requires that the help model is neither too simple, nor too complex (see Results: Choosing a second model).

The final important comparison with literature concerns that with Bayesian approaches. Bayesian approaches are centered around the combination of a prior distribution or belief with experimental data to obtain a posterior distribution. Although Bayesian calculations in practice can be done using simple calculations like the BIC, the perhaps most common approach involves Markov Chain Monte Carlo (MCMC) [51,56], and such calculations have big similarities to bootstrap approaches. One important such MCMC-based approach, which has been used in systems biology, is the Bayes Factor (BF) [8,57]. BF can be viewed as a generalization of the LHR to a Bayesian setting. In particular, this means that the likelihoods are integrated over the prior distributions of the parameters, to obtain the ratio of the marginalized distributions. Methods to do these marginalizations have been investigated in *e*.*g*. [58], and simplified calculations, not requiring the likelihood, using Approximate Bayesian Computations (ABC), are considered *e*.*g*. in [59]. This inclusion of the parameter uncertainties is important, because in systems biology the parameters are often undetermined [4], and an important continuation of this work will therefore be to compare the LHR with future extensions of the herein presented frequentist approaches to also include parameter uncertainty. On this note, it should be mentioned that we have done a small analysis to see the effect of the inclusion of such parameter uncertainties on a specific cloud by exploiting the profile likelihood (PLH) (Additional file 1: Figure S6) [60]. This small scale analysis indicates that although the results may change upon such an inclusion, the change is not big compared to other uncertainties within the method. Another way to take parameter uncertainty into account is by introducing an initial step of non-parametric bootstrapping into the bootstrap sample generation, as done e.g. in [11]. Once parameter uncertainty is taken into account in the bootstrap setting in this way, there is a striking similarity to the Bayesian Posterior Predictive (BPP) checks [11,50,61]. In BPP, the predicted distribution of future experiments is compared to the observed data. This is done by generating new data by sampling and simulating from the posterior distribution, and then comparing the resulting distribution of goodness-of-fit (GOF) with the GOF from the experimental data [11,50,61]. With all these similarities pointed out, it should also be recalled that Bayesian approaches are never identical to frequentist approaches, since frequentist approaches do not require a prior.

## Conclusions

In a bootstrap setting, it is possible to obtain joint distributions for combinations of test statistics in a more straightforward way than is possible in an analytical setting, but this possibility has previously been little explored. We here show that such combinations often do provide additional knowledge not contained in the individual tests, but that the considered simplistic combinations, like *max* and *min*, yield inconsistent, *i*.*e*. overly conservative or liberal, results (Figure 4). A new 2D approach (Figure 2), on the other hand, is only mildly conservative (Figure 5B,D), and is superior to the individual tests (Figure 5A,C). These results were obtained on both a static and dynamic case, where the truth is known (Figure 3). On the same examples, a 2D *χ*^2^ *vs* *χ*^2^ test is superior to a 2D *χ*^2^ *vs* DW test (Figure 6A,C), where the additional *χ*^2^-value comes from the ability of a second model to describe bootstrap samples from the tested model (Additional file 1: Figure S1). The 2D *χ*^2^ *vs* *χ*^2^ test is, in turn, outperformed by the 1D bootstrapped LHR (Figure 6A,C). These results are also confirmed on a previously published rejection example from insulin signaling in human fat cells, which has now been statistically resolved for the first time (Figure 8E).

Further analysis of these results show that whether or not a 2D combination is advantageous depends on a balancing between positive and negative aspects. The positive sides are found if the cloud as in Figure 1 lies in a direction non-parallel to either of the axes, and the price to exploit this is *e*.*g*. that density estimation in 2D converges more slowly, and that one cannot define one-sided or two-sided cutoffs for the tolerance regions. Similarly, the additional model only provides a benefit if it is of a rightly balanced ability to describe the data; otherwise using the additional model worsens the performance. It is because of these balancing acts between positive and negative aspects that LHR may be the better choice: if the additional model is of appropriate complexity, LHR extracts all the useful information of the *χ*^2^ *vs* *χ*^2^ plot with a one-dimensional analysis, which thus avoids the estimation of a 2D density (Figure 6A,C). This analysis thus provides a new motivation for the LHR test, which is valid in the case of non-linear and non-nested models.

In summary, these results provide useful insights into the important systems biology problem of model rejection: when to use, and when not to use, 2D approaches and additional models. These methods are to be exploited in challenging and important cases, when accuracy and power rather than computational speed are prioritized.

## Competing interests

The authors declare that they have no competing interests.

## Authors’ contributions

RJ developed, implemented, and analyzed the methods. RJ also constructed the test cases, and drafted the manuscript. PS contributed with biological models, experimental data, and interpretations. GC was the main designer of the study and co-drafted the manuscript. All authors read and approved the final manuscript.

## Supplementary Material

Additional file 1 **Supplementary material.** The PDF-file “*RJ et al. - supplementary material.pdf*” contains additional elaboration on methods used, some additional results and analysis, and discussion on some of the issues covered in this manuscript, as well as all supplementary figures referred to. Click here for file

## References

[B1] Kitano H Computational systems biology Nature 2002 420 6912 206 210 10.1038/nature01254 12432404

[B2] Cedersund G Roll J Systems biology: model based evaluation and comparison of potential explanations for given biological data FEBS J 2009 276 903 922 10.1111/j.1742-4658.2008.06845.x 19215297

[B3] Brännmark C Palmer R Glad ST Cedersund G Strålfors P Mass and information feedbacks through receptor endocytosis govern insulin signaling as revealed using a parameter-free modeling framework J Biol Chem 2010 285 20171 20179 10.1074/jbc.M110.106849 20421297 PMC2888430

[B4] Cedersund G Conclusions via unique predictions obtained despite unidentifiability–new definitions and a general method FEBS J 2012 279 18 3513 3527 10.1111/j.1742-4658.2012.08725.x 22846178

[B5] Popper KR Conjectures and Refutations: The Growth of Scientific Knowledge 2002 London: Routledge

[B6] Nyman E Brannmark C Palmer R Brugard J Nystrom FH Strålfors P Cedersund G A hierarchical whole-body modeling approach elucidates the link between in Vitro insulin signaling and in Vivo glucose homeostasis J Biol Chem 2011 286 29 26028 26041 10.1074/jbc.M110.188987 21572040 PMC3138269

[B7] Nyman E Fagerholm S Jullesson D Strålfors P Cedersund G Mechanistic explanations for counter-intuitive phosphorylation dynamics of the insulin receptor and insulin receptor substrate-1 in response to insulin in murine adipocytes FEBS J 2012 279 6 987 999 10.1111/j.1742-4658.2012.08488.x 22248283

[B8] Schmidl D Hug S Li WB Greiter MB Theis FJ Bayesian model selection validates a biokinetic model for zirconium processing in humans BMC Syst Biol 2012 6 95 10.1186/1752-0509-6-95 22863152 PMC3529705

[B9] Timmer J Müller TG Swameye I Sandra O Klingmüller U Modeling the nonlinear dynamics of cellular signal transduction Int J Bifurcation Chaos 2004 14 6 2069 2079 10.1142/S0218127404010461

[B10] Müller TG Faller D Timmer J Swameye I Sandra O Klingmüller U Tests for cycling in a signalling pathway Appl Stat 2004 53 4 557 558

[B11] Wagenmakers EJ Ratcliff R Gomez P Iverson GJ Assessing model mimicry using the parametric bootstrap J Math Psychol 2004 48 28 50 10.1016/j.jmp.2003.11.004

[B12] Melykuti B August E Papachristodoulou A El-Samad H Discriminating between rival biochemical network models: three approaches to optimal experiment design BMC Syst Biol 2010 4 38 10.1186/1752-0509-4-38 20356406 PMC2873315

[B13] Roberts MA August E Hamadeh A Maini PK McSharry PE Armitage JP Papachristodoulou A A model invalidation-based approach for elucidating biological signalling pathways, applied to the chemotaxis pathway in R. sphaeroides BMC Syst Biol 2009 3 105 10.1186/1752-0509-3-105 19878602 PMC2783038

[B14] Ljung L System Identification (2nd Ed.): Theory for the User 1999 Upper Saddle River, NJ, USA: Prentice Hall PTR

[B15] Vuong QH Likelihood ratio tests for model selection and non-nested hypotheses Econometrica 1989 57 2 307 333 10.2307/1912557

[B16] Cox DR Tests of separate families of hypotheses Proc 4th Berkeley Symp Math Stat Probab 1961 1 105 123

[B17] Cox DR Further results on tests of separate families of hypotheses J R Stat Soc Series B (Methodol) 1962 24 2 406 424

[B18] Sheskin DJ Handbook of Parametric and Nonparametric Statistical Procedures 2011 London: A Chapman & Hall book, Chapman & Hall/CRC

[B19] Chernoff H On the distribution of the likelihood Ratio Ann Math Stat 1954 25 3 573 587 10.1214/aoms/1177728725

[B20] Chant D On asymptotic tests of composite hypotheses in nonstandard conditions Biometrika 1974 61 2 291 298 10.1093/biomet/61.2.291

[B21] Miller JJ Asymptotic properties of maximum likelihood estimates in the mixed model of the analysis of variance Ann Stat 1977 5 4 746 762 10.1214/aos/1176343897

[B22] Shapiro A Asymptotic distribution of test statistics in the analysis of moment structures under inequality constraints Biometrika 1985 72 1 133 144 10.1093/biomet/72.1.133

[B23] Self SG Liang K-Y Asymptotic properties of maximum likelihood estimators and likelihood ratio tests under nonstandard conditions J Am Stat Assoc 1987 82 398 605 610 10.1080/01621459.1987.10478472

[B24] Kanji GK 100 Statistical Tests 2006 Thousand Oaks, California, US: SAGE Publications

[B25] Williams DA Discrimination between regression models to determine the pattern of enzyme synthesis in synchronous cell cultures Biometrics 1970 26 23 32 10.2307/2529041 5437360

[B26] Efron B Bootstrap methods: another look at the Jackknife Ann Stat 1979 7 1 1 26 10.1214/aos/1176344552

[B27] Efron B The Jackknife, the Bootstrap, and Other Resampling Plans (CBMS-NSF Regional Conference Series in Applied Mathematics) 1987 Montpelier, Vermont, USA: Society for Industrial Mathematics

[B28] Kerr MK Churchill GA Bootstrapping cluster analysis: assessing the reliability of conclusions from microarray experiments Proc Natl Acad Sci USA 2001 98 16 8961 8965 10.1073/pnas.161273698 11470909 PMC55356

[B29] Kirk PD Stumpf MP Gaussian process regression bootstrapping: exploring the effects of uncertainty in time course data Bioinformatics 2009 25 10 1300 1306 10.1093/bioinformatics/btp139 19289448 PMC2677737

[B30] Felsenstein J Confidence limits on phylogenies: an approach using the bootstrap Evolution 1985 39 4 783 791 10.2307/2408678 28561359

[B31] Efron B Halloran E Holmes S Bootstrap confidence levels for phylogenetic trees Proc Natl Acad Sci USA 1996 93 14 7085 7090 10.1073/pnas.93.14.7085 8692949 PMC38940

[B32] Lanfear R Bromham L Statistical tests between competing hypotheses of Hox cluster evolution Syst Biol 2008 57 5 708 718 10.1080/10635150802430079 18853358

[B33] Hinde J Fahrmeir L Choosing between nonnested models: a simulation approach Advances in GLIM and Statistical Modelling. Proceedings of the Glim92 Conference 1992 Munich, Germany: Springer-Verlag

[B34] National-Research-Council-(US) Combining Information: Statistical Issues and Opportunities for Research. Contemporary statistics 1992 Washington DC: National Academy Press

[B35] Bailey TL Gribskov M Combining evidence using p-values: application to sequence homology searches Bioinformatics 1998 14 1 48 54 10.1093/bioinformatics/14.1.48 9520501

[B36] Louv WC Littell RC Combining one-sided binomial tests J Am Stat Assoc 1986 81 394 550 554 10.1080/01621459.1986.10478303

[B37] Wilkinson B A statistical consideration in psychological research Psychol Bull 1951 48 3 156 158 14834286 10.1037/h0059111

[B38] Hubner K Sahle S Kummer U Applications and trends in systems biology in biochemistry FEBS J 2011 278 16 2767 2857 10.1111/j.1742-4658.2011.08217.x 21707921

[B39] Heinrich R Schuster S The Regulation of Cellular Systems 1996 London: Chapman & Hall

[B40] MATLAB Version 7.13.0.564 (R2011b) 2011 Natick, Massachusetts: The MathWorks Inc.

[B41] Schmidt H Jirstrand M Systems biology toolbox for MATLAB: a computational platform for research in systems biology Bioinformatics 2006 22 514 515 10.1093/bioinformatics/bti799 16317076

[B42] Silverman BW Density Estimation for Statistics and Data Analysis. Monographs on applied probability and statistics 1986 London: Chapman and Hall

[B43] Cao Y Bivariant Kernel Density Estimation (V2.0) 2008 The MathWorks, Inc http://www.mathworks.com/matlabcentral/fileexchange/19280-bivariant-kernel-density-estimation-v2-0/content/gkde2.m

[B44] Hastie TJ Tibshirani RJ Friedman JJH The Elements of Statistical Learning: Data Mining, Inference, and Prediction. Springer Series in Statistics 2001 Munich, Germany: Springer

[B45] Cedersund G Roll J Ulfhielm E Danielsson A Tidefelt H Strålfors P Model-based hypothesis testing of key mechanisms in initial phase of insulin signaling PLoS Comput Biol 2008 4 1000096 10.1371/journal.pcbi.1000096 PMC2424138 18551197

[B46] Akaike H A new look at the statistical model identification IEEE Trans Automatic Control 1974 19 6 716 723 10.1109/TAC.1974.1100705

[B47] Akaike H Eykoff P Modern development of statistical methods Trends and Progress in System Identification 1981 New York: Pergamon Press

[B48] Neyman J Pearson ES On the use and interpretation of certain test criteria for purposes of statistical inference Biometrika 1928 20A 1-2 175 240 10.1093/biomet/20A.1-2.175

[B49] Godfrey LG On the asymptotic validity of a bootstrap method for testing nonnested hypotheses Econ Lett 2007 94 3 408 413 10.1016/j.econlet.2006.08.031

[B50] Bollback JP Bayesian model adequacy and choice in phylogenetics Mol Biol Evol 2002 19 7 1171 1180 10.1093/oxfordjournals.molbev.a004175 12082136

[B51] Box GEP Tiao GC Bayesian Inference in Statistical Analysis. Wiley Classics Library 2011 New York: Wiley

[B52] Apgar JF Toettcher JE Endy D White FM Tidor B Stimulus design for model selection and validation in cell signaling PLoS Comput Biol 2008 4 2 30 10.1371/journal.pcbi.0040030 PMC2323406 18282085

[B53] Dochain D Vanrolleghem P Dynamical Modelling and Estimation in Wastewater Treatment Processes 2001 London: IWA Publishing

[B54] Wilks SS The large-sample distribution of the likelihood ratio for testing composite hypotheses Ann Math Stat 1938 9 1 60 62 10.1214/aoms/1177732360

[B55] Hall P Wilson SR Two guidelines for bootstrap hypothesis testing Biometrics 1991 47 2 757 762 10.2307/2532163

[B56] Geyer CJ Practical Markov chain Monte Carlo Stat Sci 1992 7 4 473 483 10.1214/ss/1177011137

[B57] Xu TR Vyshemirsky V Gormand A von Kriegsheim A Girolami M Baillie GS Ketley D Dunlop AJ Milligan G Houslay MD Kolch W Inferring signaling pathway topologies from multiple perturbation measurements of specific biochemical species Sci Signal 2010 3 134 20 10.1126/scisignal.2000517 20234003

[B58] Vyshemirsky V Girolami MA Bayesian ranking of biochemical system models Bioinformatics 2008 24 6 833 839 10.1093/bioinformatics/btm607 18057018

[B59] Toni T Welch D Strelkowa N Ipsen A Stumpf MP Approximate Bayesian computation scheme for parameter inference and model selection in dynamical systems J R Soc Interface 2009 6 31 187 202 10.1098/rsif.2008.0172 19205079 PMC2658655

[B60] Raue A Kreutz C Maiwald T Bachmann J Schilling M Klingmuller U Timmer J Structural and practical identifiability analysis of partially observed dynamical models by exploiting the profile likelihood Bioinformatics 2009 25 15 1923 1929 10.1093/bioinformatics/btp358 19505944

[B61] Rubin DB Bayesianly justifiable and relevant frequency calculations for the applied statistician Ann Stat 1984 12 4 1151 1172 10.1214/aos/1176346785

