# Supplementary material for: Combining test statistics and models in bootstrapped model rejection: it is a balancing act
Source: BMC Syst Biol. 2014 Apr 17;8:46. doi: 10.1186/1752-0509-8-46 (PMC4022267; doi:10.1186/1752-0509-8-46)
Supplement: Additional file 1 — Supplementary material. The PDF-file “RJ et al. - supplementary material.pdf” contains additional elaboration on methods used, some additional results and analysis, and discussion on some of the issues covered in this manuscript, as well as all supplementary figures referred to. [file 1752-0509-8-46-S1.pdf]

Combining test statistics and models in bootstrapped model rejection: it is a balancing act

Supplementary Material

Rikard Johansson, Peter Strålfors , Gunnar Cedersund*

Email: GC: gunnar.cedersund@liu.se;

*Corresponding author

Methods

The methods included in this section are all briefly described in the main paper but in less detail, due to large overlap in implementation with other methods already described. Now follows a full description of these methods.

Tests involving two models

Bootstrapped two-dimensional χ^2 vs χ^2 test

This test was briefly outlined in the Methods section of the main document. It is explained in more detail here. This method expands the one-dimensional χ^2 bootstrap approach by considering an extra dimension, but this time not with the DW test statistic but with a second χ^2-test statistic corresponding to a second model. The method steps are summarized in Supplementary Figure S1.

Algorithm

Given two models, $\mathcal{M}_1$ and $\mathcal{M}_2$, an experimental data set $\mathcal{Z}$, a test statistic $\mathcal{T}$ (here exemplified with the χ^2 test statistic, $\mathcal{T}_{\chi^2}$), and a significance level α:

1. Fit both models to the original data set $\mathcal{Z}$ and let $\widehat{\theta}_{M_1}$ and $\widehat{\theta}_{M_2}$ denote the estimated parameter vectors. Calculate the statistics $\mathcal{T}_{\chi^2}^{\mathcal{M}_1}(\mathcal{Z})$ and $\mathcal{T}_{\chi^2}^{\mathcal{M}_2}(\mathcal{Z})$ according to Equation 6 in the main document.

2. Use $\mathcal{M}_1(\widehat{\theta}_{M_1})$ and $\mathcal{M}_2(\widehat{\theta}_{M_2})$ to generate two sets of bootstrap samples. These sets are denoted $\mathcal{B}_1$ and $\mathcal{B}_2$, respectively.

3. Fit $\mathcal{M}_1$ and $\mathcal{M}_2$ to each bootstrap sample, b, in $\mathcal{B}_1$ and $\mathcal{B}_2$. This results in four sets of χ^2-values, which we divide into two clouds:

   - $\mathcal{C}_1$, cloud 1, consisting of $\mathcal{T}_{\chi^2}^{\mathcal{M}_1}(\mathcal{B}_1)$ and $\mathcal{T}_{\chi^2}^{\mathcal{M}_2}(\mathcal{B}_1)$, obtained by fitting both models to each $b \in \mathcal{B}_1$.
   - $\mathcal{C}_2$, cloud 2, consisting of $\mathcal{T}_{\chi^2}^{\mathcal{M}_1}(\mathcal{B}_2)$ and $\mathcal{T}_{\chi^2}^{\mathcal{M}_2}(\mathcal{B}_2)$, obtained by fitting both models to each $b \in \mathcal{B}_2$.

4. Estimate the two-dimensional empirical distributions (see section Two-dimensional density estimation in main document) $\rho_1(\mathcal{C}_1)$ and $\rho_2(\mathcal{C}_2)$. Let $\rho_1(\mathcal{Z}) := \rho_1(\mathcal{T}_{\chi^2}^{\mathcal{M}_1}(\mathcal{Z}), \mathcal{T}_{\chi^2}^{\mathcal{M}_2}(\mathcal{Z}))$ and $\rho_2(\mathcal{Z}) := \rho_2(\mathcal{T}_{\chi^2}^{\mathcal{M}_1}(\mathcal{Z}), \mathcal{T}_{\chi^2}^{\mathcal{M}_2}(\mathcal{Z}))$ denote the two obtained densities at the coordinate corresponding to the χ^2-values of the original data set $\mathcal{Z}$. For each distribution, we define the cutoff plane as the equidensity contour that goes through $\rho_1(\mathcal{Z})$ or $\rho_2(\mathcal{Z})$ respectively.

5. Using the two-dimensional distributions, calculate the p-value for each model

$$p_{\chi^2-\chi^2}^{\mathcal{M}_1} := \int_{\rho_1 < \rho_1(\mathcal{Z})} \rho_1(\mathcal{T}_{\chi^2}^{\mathcal{M}_1}(\mathcal{B}_1), \mathcal{T}_{\chi^2}^{\mathcal{M}_2}(\mathcal{B}_1)) d\mathcal{T}_{\chi^2}^{\mathcal{M}_1} d\mathcal{T}_{\chi^2}^{\mathcal{M}_2}$$

$$p_{\chi^2-\chi^2}^{\mathcal{M}_2} := \int_{\rho_2 < \rho_2(\mathcal{Z})} \rho_2(\mathcal{T}_{\chi^2}^{\mathcal{M}_1}(\mathcal{B}_2), \mathcal{T}_{\chi^2}^{\mathcal{M}_2}(\mathcal{B}_2)) d\mathcal{T}_{\chi^2}^{\mathcal{M}_1} d\mathcal{T}_{\chi^2}^{\mathcal{M}_2}$$

   If $p_{\chi^2-\chi^2}^{\mathcal{M}_1} < \alpha$, then $\mathcal{M}_1$ should be rejected, and similarly if $p_{\chi^2-\chi^2}^{\mathcal{M}_2} < \alpha$, $\mathcal{M}_2$ should be rejected. This will result in that one, both, or neither of the hypotheses are rejected.

Bootstrapped log likelihood ratio test

This test was briefly outlined in the Methods section of the main document. It is explained in more detail here. The procedure for this method is very similar to the 2D χ^2 *vs* χ^2 test described above. The first 3 method steps are identical to this method, with the exception that the resulting clouds in step 3 are the 1D distribution of the χ^2 differences. All steps are included below for easy reference.

Algorithm

Given two models, $\mathcal{M}_1$ and $\mathcal{M}_2$, an experimental data set $\mathcal{Z}$, a test statistic $\mathcal{T}$ (here exemplified with the χ^2 test statistic, $\mathcal{T}_{\chi^2}$), and a significance level α:

1. Fit both models to the original data set $\mathcal{Z}$ and let $\widehat{\theta}_{M_1}$ and $\widehat{\theta}_{M_2}$ denote the estimated parameter vectors. Calculate the statistics $\mathcal{T}_{\chi^2}^{\mathcal{M}_1}(\mathcal{Z})$ and $\mathcal{T}_{\chi^2}^{\mathcal{M}_2}(\mathcal{Z})$ according to Equation 6 in the main document.

2. Use $\mathcal{M}_1(\widehat{\theta}_{M_1})$ and $\mathcal{M}_2(\widehat{\theta}_{M_2})$ to generate two sets of bootstrap samples. These sets are denoted $\mathcal{B}_1$ and $\mathcal{B}_2$, respectively.

3. Fit $\mathcal{M}_1$ and $\mathcal{M}_2$ to each bootstrap sample, b, in $\mathcal{B}_1$ and $\mathcal{B}_2$. This results in four sets of χ^2-values, which we divide into two clouds:

   - $\mathcal{C}_1 := \mathcal{T}_{\chi^2}^{\mathcal{M}_1}(\mathcal{B}_1)$ -$\mathcal{T}_{\chi^2}^{\mathcal{M}_2}(\mathcal{B}_1)$, the empirical distribution of $\mathcal{T}_{LHR}$ when $\mathcal{H}_0 = \mathcal{M}_1$.
   - $\mathcal{C}_2 := \mathcal{T}_{\chi^2}^{\mathcal{M}_1}(\mathcal{B}_2)$ - $\mathcal{T}_{\chi^2}^{\mathcal{M}_2}(\mathcal{B}_2)$,the empirical distribution of $\mathcal{T}_{LHR}$ when $\mathcal{H}_0 = \mathcal{M}_2$.

4. The above resulting distributions of LHR-values (χ^2-differences) are evaluated using MATLAB and the empirical cumulative distribution function, *ecdf*. A p-value, under each $\mathcal{H}_0$, is obtained by considering a two-tailed distribution and interpolating the value of the cumulative empirical distribution at the coordinate corresponding to the LHR-value of the original data set.

Results and Discussion

This section contains some additional results and elaboration on issues mentioned in the main paper.

On simplistic combinations

In the main paper some simplistic combinations of p-values were proposed, and then analyzed with respect to their type I error rate (Figure 4, main paper). Supplementary Figure S7 shows the ROC curves corresponding to these simplistic combinations. Interestingly, they exhibited an AUC that was comparative to, and sometimes even higher than, the AUC for individual test statistics. Despite this, it was decided not to proceed with these combinations due to the unsound type I error rate behavior demonstrated in Figure 4 (main paper). It should be pointed out that for a real example, the TPR and FPR cannot be assessed, and therefore the level of liberalness or conservativeness cannot be quantified and accounted for. One would therefore have no way of trusting an obtained p-value.

On choosing the second model

In the main paper we showed how using a bad second model would reduce the performance of the tests involving two models. Figure 7D in the main paper illustrates how using a hyper-flexible model collapses

the cloud to one axis, which nullifies any possibly advantage available by using a second dimension and/or a second model. Conversely, a help model which would collapse the cloud to the other axis should provide a similar outcome. Thus a secondary model that always gives a very high cost, in contrast to the hyper-flexible model, is a natural choice. We reasoned that a dummy model that approximated the data with a constant straight line would be a good candidate and used this on the same test cases as in the main paper. Interestingly, we here for the first time found a major difference between the static and dynamic test case.

For the static case, the results were as expected of a non-informative help model. In the ROC-analysis (Supplementary Figure S5), the 2D χ^2 *vs* χ^2 (purple, dash dotted line) performed no better than an ordinary 1D bootstrapped χ^2-test (red, solid line). Since these two ROC curves have approximately the same AUC however, the χ^2 *vs* χ^2 method still managed to capture the information contained in the informative dimension (the χ^2-values of the realistic model). The 1D LHR method on the other hand, was rendered completely uninformative when using this dummy model (orange, dash dotted line lies parallel to the identity line). Unlike the 2D analysis, this method does not retain the informative dimension, which instead is squelched in the noise of the large residuals of the constant model (the uninformative dimension). This should be contrasted to the hyper-flexible model where the LHR collapses to a two-sided χ^2-test (Figure 7E, Main paper).

The situation is more complex for the dynamic test cases. Here it actually turns out that the extra dimension granted by the dummy model is not uninformative. As can be seen in Supplementary Figure S2C, the χ^2-values for the dummy model fitted to $\mathcal{M}_{D1}$ bootstraps typically lie between 80-240. For $\mathcal{M}_{D2}$ bootstraps, however, the χ^2-values of the dummy model typically lie between 130-300 (Supplementary Figure S2D). $\mathcal{M}_{D1}$ and $\mathcal{M}_{D2}$ thus have very different, and almost non-overlapping distributions of the χ^2-cost for the constant dummy model. This is a result of the chosen model structures. As can be seen in Supplementary Figure S2B, $\mathcal{M}_{D1}$ will tend to be more centered around its mean value than $\mathcal{M}_{D2}$. The gray area in Supplementary Figure S2B depicts the only region of the x-axis where $\mathcal{M}_{D1}$ contributes with a higher cost for the dummy model than $\mathcal{M}_{D2}$. Since the gray interval is much smaller than the total interval, $\mathcal{M}_{D1}$, data point for data point, typically has a lower cost and it is this behavior that results in the almost non-overlapping χ^2-distributions of the dummy model. This means that the distribution of the (artificial) data around the mean value will in itself be an informative statistic with

enough information to discriminate between the two models in some noise realizations. Consequently, Supplementary Figure S2A shows how the 2D χ^2 *vs* χ^2 (purple, dash dotted line) performs slightly better than the single 1D bootstrapped χ^2-test (red, solid line), and the 1D LHR (orange, dash dotted line) is no longer completely uninformative (it no longer lies parallel to the identity line as was the case in the static example, Supplementary Figure S5). However, the LHR is still worse off than just simply doing the 1D χ^2-test. All in all, this illustrates that while it may be hard in advance to determine what will be a poor help model, and what will be a good help model, an inspection of the distribution of the clouds will give a good indication of which dimensions that have information, and, by looking at the tilt of the clouds, whether this information benefits from a 2D analysis, as compared to investigating one dimension at a time.

On parameter uncertainty and limitations of the density estimator in 2D

One possible issue with the methods used herein is the usage of $\widehat{\theta}$ when generating bootstrap samples from a model (see step 1-2 in presented method algorithms). In the dynamic setting of ODE models, $\widehat{\theta}$, or the Maximum Likelihood Estimator (MLE), is a single parameter value obtained from an optimization. However, because many of the model parameters have to be estimated in this way and cannot be uniquely determined, there is an uncertainty in the parameter values, and some parameters may even be structurally or practically unidentifiable. Regardless, taking parameter uncertainty into account was something that could potentially affect the shape of the clouds and we therefore checked this on the insulin signaling case. Using the profile likelihood (PLH), we estimated the uncertainty of the $\mathcal{M}_{i,c}$ model parameters and created a set of 100 parameters vectors from these profiles from which we generated the bootstrap samples. This set of 100 parameters was constructed by sampling each of the 10 parameter profiles of the model, 10 times each, log-uniformly from highest to lowest value.

Supplementary Figure S6A-B shows how the overall shape and geometry of the cloud seems unaffected by the inclusion of parameter uncertainty (the green circles and overall mass of the density distributions are indistinguishable). Nevertheless, when estimating the density and calculating the p-value for the observed experimental data, there is a 5-fold discrepancy between the two cases, $p = 4.1 * 10^{-3}$ and $p = 8.6 * 10^{-4}$ for the PLH and MLE cases respectively (Supplementary Figure S6C-D). However, since the point of interest is in a region with low density in both cases, and because in one cloud (B), a realization is just in the vicinity of the red dot, but not so in the other cloud (A), we wanted to put this uncertainty in relation to the density estimation uncertainty for these low density regions.

We investigated the sensitivity of the density estimation in low density-regions to new cloud realizations by subdividing the total clouds into 5 separate sub-clouds each. We then estimated the density at the point of the experimental data (Supplementary Figure S6A-B, red dots), and some chosen points with various degree of density (blue triangles). The results are shown in Supplementary Figure S6C-D. The figures show the p-value estimation of a hypothetical data point (blue triangles, red dot) at the indicated x- and y-coordinates. The first two columns, x and y, of each table corresponds to these coordinates. The third column, *p-tot*, corresponds to the p-value estimate obtained when using the whole cloud (cloud size 10^4), while the 5 following columns, *p-s1* to *p-s5*, correspond to the 5 different sub-cloud realizations (cloud size 2000). The last column, *pmax/pmin*, shows the ratio between the highest and lowest of these sub-cloud p-value estimates at the indicated coordinate. Each row correspond to a different x- and y-coordinate, chosen to represent various degrees of density, including the experimental data. Supplementary Figure S6A shows the result for the MLE based approach to generating bootstrap samples, whereas Supplementary Figure S6B shows the results for the PLH based approach. As can be seen, due to the low number of points when considering these low-density regions, there is a high variation between different sub-clouds within each method. For instance, at the coordinates of the experimental data, the MLE has a *pmax/pmin* ratio of 15, and the PLH approach 950. While this uncertainty is bigger than the observed 5-fold difference in p-values of the total clouds, the sub-clouds are in contrast smaller, and a higher variability is expected as when compared to the full cloud. However, the variability of the density estimation of the full cloud (cloud size 10^4) has not been quantified. Further analysis is warranted before deciding either way whether this discrepancy between the MLE and PLH approach is consistent.

On the data generating process noise level

In the methods section of the main paper the noise levels used for creating the type I error curves and ROC curves were specified: σ= 0.5 and 0.75 for the the static and dynamic test cases, respectively. Varying the degree of noise in the data generating process does effect the outcome of the tests, but the only effect is to makes it harder, or easier, to discriminate between models. In other words, such variations in the noise level do not change which of the methods that performs better. Supplementary Figure S8A-C shows that when the noise level increases, the ROC curves approach the identity line, but that their order (and therefore their ranking by AUC) is kept. Put in another way, the number of true positives goes down, and the number of false positives goes up, *i.e.* more mistakes are made, but the methods that makes the

most or least mistakes are still the same. Why the difficulty increases is illustrated on a number of example clouds from the 2D χ^2 vs χ^2 method (Supplementary Figure S8D-F). When the noise level increases, the false model becomes increasingly equally good as the true model at describing data generated by the true model. This is because the noise term in Equation (4) in the main paper will dominate the expression, and any true signal will disappear. As a result, the clouds will tend to lie along the identify line (45 degree angle) for higher levels of noise.

Supplementary Figures

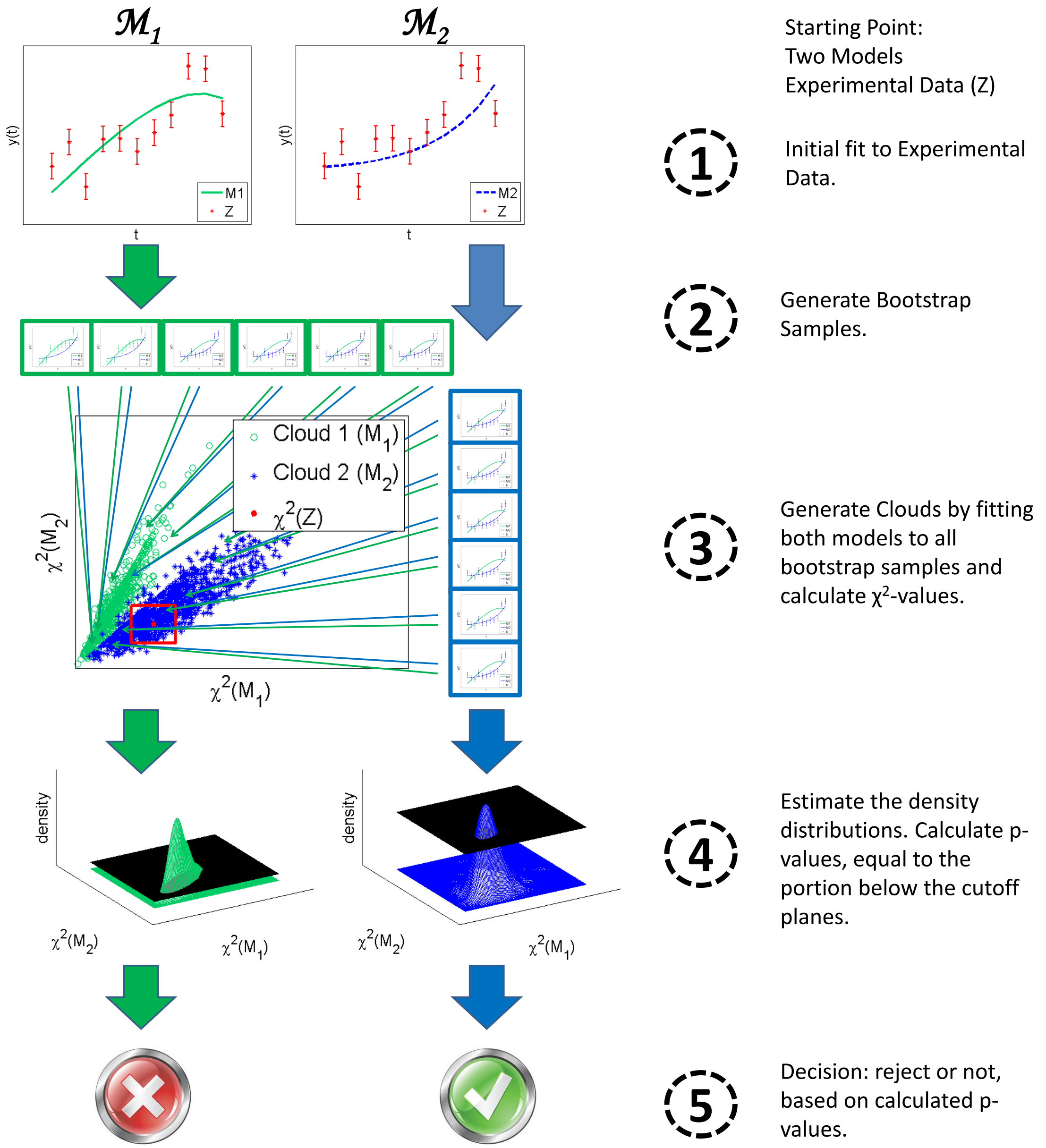

Supplementary Figure S1: A graphical summary of the proposed method steps for a 2D χ^2 vs χ^2 bootstrap approach. Encircled numbers correspond to the steps described in the model algorithm. The starting point is some data set, $\mathcal{Z}$, and two models. These two models are fitted against the experimental data and then used to generate two sets of bootstraps samples. Next, both models are fitted against all bootstrap samples in each set. The corresponding two-dimensional clouds are used to generate density estimations of both hypotheses. We define the cutoff planes as the equidensity contour lines, for each cloud, that goes through the coordinates of the experimental data (red square). The corresponding p-values are equal to the portion of the distribution below the plane. The p-values are then used for decision on whether or not to reject either model.

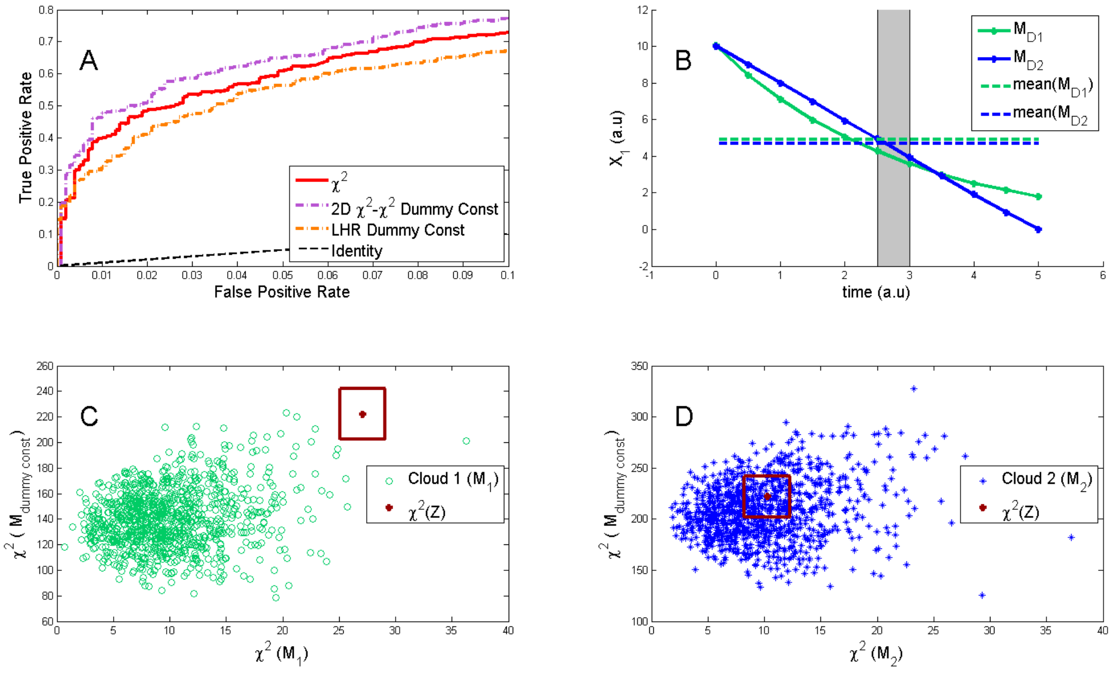

Supplementary Figure S2: Analysis of using a constant as choice of second model. (A) ROC curves for the dynamic case when using a constant as a dummy model. Figure shows ordinary bootstrapped χ^2-test (red, solid), the 2D χ^2 vs χ^2 test (purple, dash dotted), and the 1D LHR test (orange, dash dotted). Panel (B) shows how the distribution of the data set around its mean value can hold information. Shown are average model bootstrap series for $\mathcal{M}_{D1}$ (green, solid) and $\mathcal{M}_{D2}$ (blue, solid). Both models have the same mean output (green dashed, and blue dashed respectively). Notice the imposed offset between the lines to avoid superposition. The gray area depicts the region where $\mathcal{M}_{D1}$ has a greater deviation from its mean value than $\mathcal{M}_{D2}$ (the distance between the green solid line and the green dashed line is greater than the distance between the blue solid line and the blue dashed line). Outside this region $\mathcal{M}_{D1}$ is closer to its mean and have a lower χ^2-cost for the dummy model. Panels (C,D) show how the distribution of the cost for a constant dummy model will differ during the bootstrap analysis ($\mathcal{M}_{D2}$ used as the true model). Because this tendency of $\mathcal{M}_{D1}$ to lie more centered around its mean than $\mathcal{M}_{D2}$, it will in general have lower cost for the dummy model. This is reflected in the vertical shift of dummy χ^2-distributions between the two clouds. The $\mathcal{M}_{D1}$ cloud lies in the range of 80-150, whereas the $\mathcal{M}_{D2}$ cloud lies in the range of 130-300. All in all, this explains while the use of a Constant Dummy Model is an informative dimension, as revealed by the analysis shown in (A).

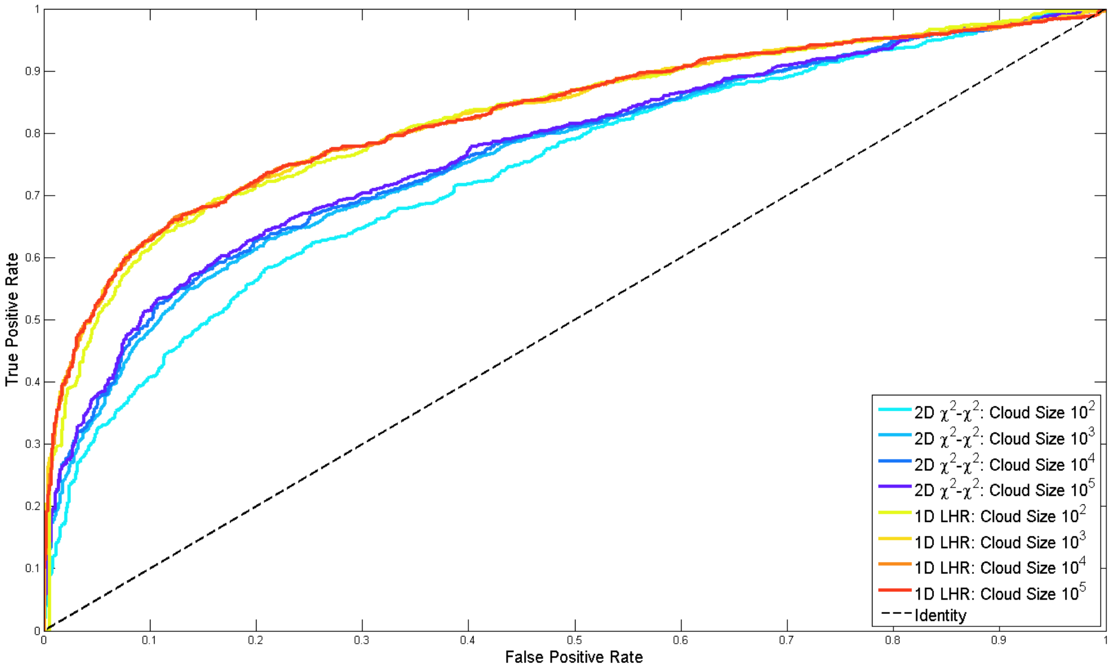

Supplementary Figure S3: Effect of varying cloud size on ROC performance in the static case for the 1D LHR analysis and the 2D χ^2 *vs* χ^2 test. The bootstrapped LHR (yellow to red lines) converges already at moderate cloud sizes. The bootstrapped 2D χ^2 *vs* χ^2 test (cyan to blue lines) requires larger cloud sizes to reach convergence.

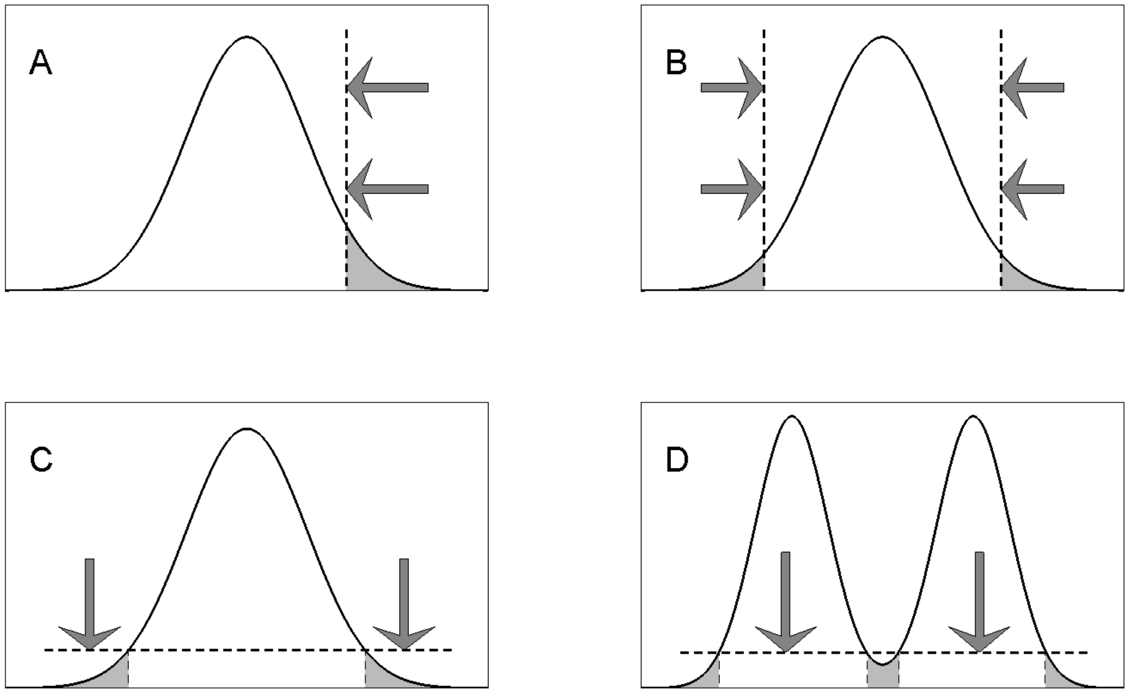

Supplementary Figure S4: Empirical tolerance intervals.(A) the standard way of defining a 1D right-tailed, or conversely left-tailed tolerance interval. It can be visualized by sliding a "cutoff bar" from infinity until the cumulative density reaches your specified cutoff value (Gray area, *e.g.* 5 %). (B) a two tailed tolerance interval can be defined in the same way, this time sliding 2 bars from both directions until the sum of the two tail areas reaches your specified cutoff. (C) alternatively one can slide a horizontal bar until the tolerance interval consists only of the interval of 95% highest density. If the distribution is symmetrical this interval will be the same as in (B). (D) if the distribution is bimodal, or even more complex, the same method as in (C) will result in a fragmented, *i.e.* discontinuous tolerance interval. The 2D equivalent of the definition used in (C,D) is the one used in this paper for the methods using an additional dimension.

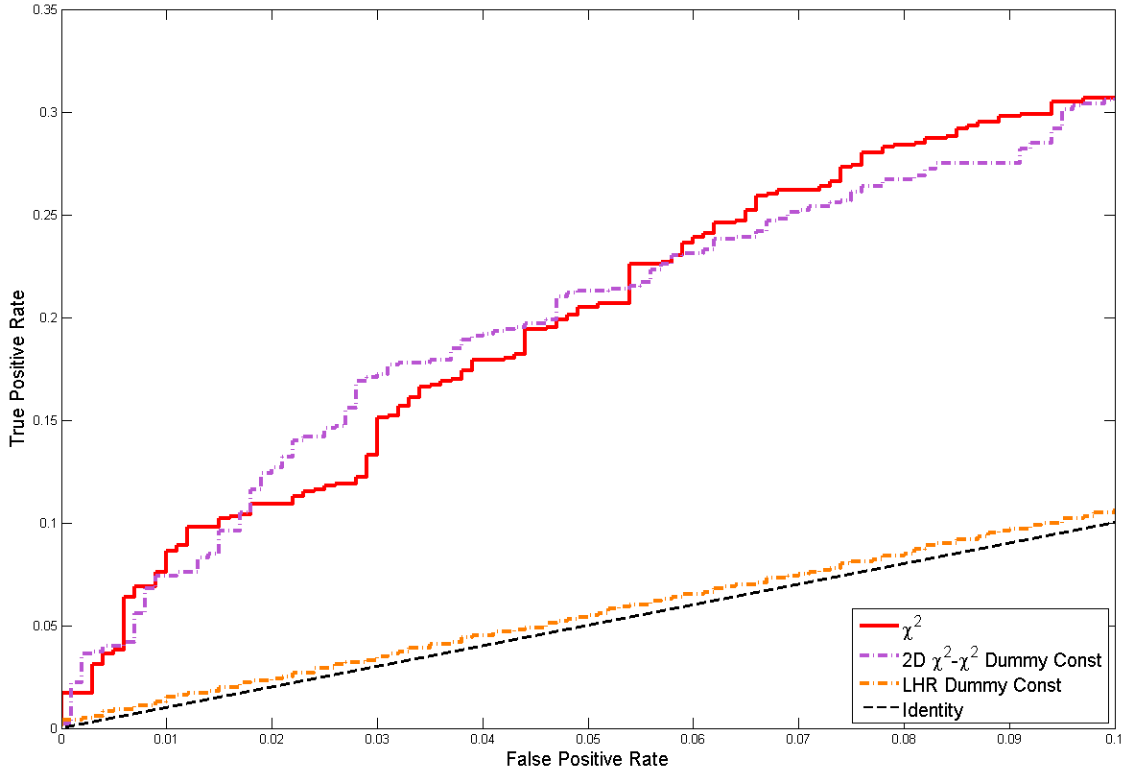

Supplementary Figure S5: Analysis of using a constant as choice of second model. ROC curves for the *static* case when using a constant as a dummy model. Figure shows ordinary bootstrapped χ^2-test (red, solid), the 2D χ^2 *vs* χ^2 test (purple, dash dotted), and the 1D LHR test (orange, dash dotted).

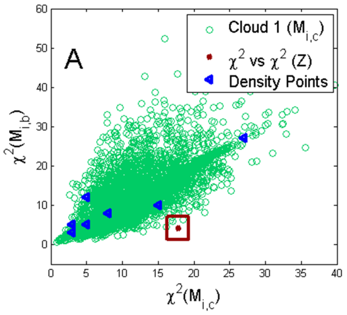

C (MLE)

x	y	p-tot	p-s1	p-s2	p-s3	p-s4	p-s5	pmax/pmin
17.8	4.1	8.6E-04	1.2E-03	1.5E-03	2.4E-03	8.6E-04	1.5E-04	15.9
27	27	1.1E-02	8.9E-03	1.5E-02	1.2E-02	1.4E-02	6.1E-03	2.5
5	12	5.6E-02	7.9E-02	5.0E-02	5.2E-02	5.2E-02	7.4E-02	1.6
15	10	1.4E-01	1.7E-01	1.6E-01	1.6E-01	1.5E-01	1.8E-01	1.2
3	5	2.8E-01	3.4E-01	3.8E-01	3.4E-01	3.1E-01	3.4E-01	1.2
3	3	4.2E-01	3.9E-01	4.4E-01	4.0E-01	3.6E-01	4.4E-01	1.2
5	5	7.7E-01	7.6E-01	7.9E-01	7.7E-01	7.6E-01	7.8E-01	1.0
8	8	9.8E-01	9.9E-01	9.9E-01	9.8E-01	9.8E-01	9.8E-01	1.0

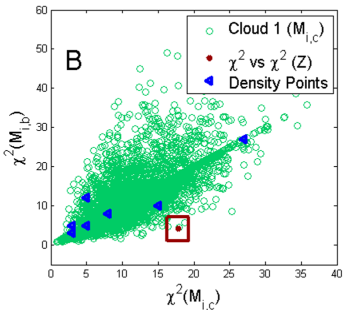

D (PLH)

x	y	p-tot	p-s1	p-s2	p-s3	p-s4	p-s5	pmax/pmin
17.8	4.1	4.1E-03	3.2E-03	1.7E-04	1.4E-05	3.4E-04	1.3E-02	950.1
27	27	2.2E-02	1.0E-02	2.0E-02	5.8E-03	3.0E-02	2.6E-02	5.1
5	12	5.9E-02	6.8E-02	8.3E-02	5.6E-02	5.1E-02	6.0E-02	1.6
15	10	1.4E-01	1.7E-01	1.8E-01	1.5E-01	1.5E-01	1.8E-01	1.2
3	5	2.6E-01	3.3E-01	3.6E-01	3.1E-01	3.4E-01	3.4E-01	1.2
3	3	4.1E-01	4.1E-01	3.9E-01	3.7E-01	4.0E-01	4.2E-01	1.1
5	5	8.2E-01	7.6E-01	8.3E-01	7.5E-01	8.5E-01	7.7E-01	1.1
8	8	9.7E-01	9.8E-01	9.8E-01	9.7E-01	9.7E-01	9.7E-01	1.0

Supplementary Figure S6: Effects of parameter uncertainty and sub-cloud realizations on p-value estimates. (A) depicts the same cloud (green circles) as in Figure 8D where the bootstrap samples have been generated using the Maximum Likelihood Estimate (MLE). Also included is the χ^2-values for the experimental data (red star and box) and some chosen points for density estimation (blue triangles). In panel (B) the bootstrap samples have been generated from a set of acceptable parameters, obtained via profile likelihood analysis (PLH). This does not seem to affect the overall shape of the cloud, although further analysis on this issue is warranted. Panel (C) is a table over the p-value estimates for the MLE cloud and how they are affected by looking at sub-clouds. The table gives the p-values at the given x and y coordinates for the total cloud, *p-tot*, and five different sub-clouds, *p-s1* to *p-s5*. Also shown is the ratio between the largest and smallest of the sub-cloud estimates. The x and y coordinates correspond to the red star and the blue triangles in (A) and (B). As can be seen, p-value estimates at contour lines along lower densities are more sensitive to new noise realizations. This is due to the fewer number of points available for the density estimator in the periphery of the cloud. In (D) the same analysis is made on the PLH cloud. Noticeably the p-value corresponding to the experimental data (red star) is approximately 5-fold higher as compared to the MLE-cloud when using the total clouds ($p = 4.1 * 10^{-3}$ and $p = 8.6 * 10^{-4}$ respectively. However, since the *intra-cloud* variability is so high at these low densities, this effect cannot be safely confirmed, and further analysis is warranted.

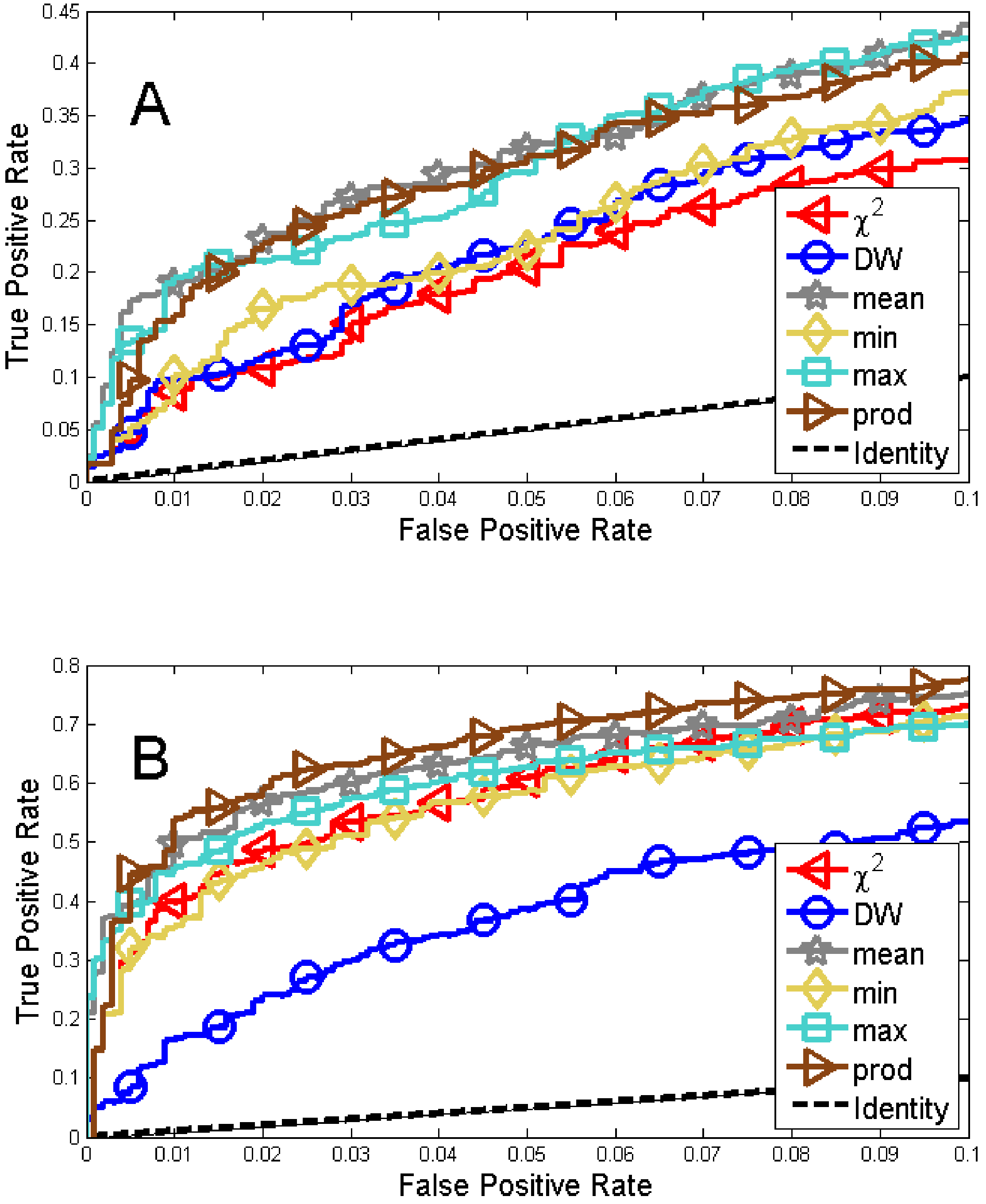

Supplementary Figure S7: ROC curves for the single test statistics and their simplistic combinations corresponding to the type I error rate plots in Figure 4 in the main paper. (A) Static example. (B) Dynamic example. All methods combining the test statistics do have an AUC that is comparative to, and sometimes even higher than, the AUC for the individual test statistics. While this is indicative of a good test, we nonetheless discarded the simplistic combinations from further analysis, due to the unsound behavior shown when studying the type I error rate (Figure 4, main paper).

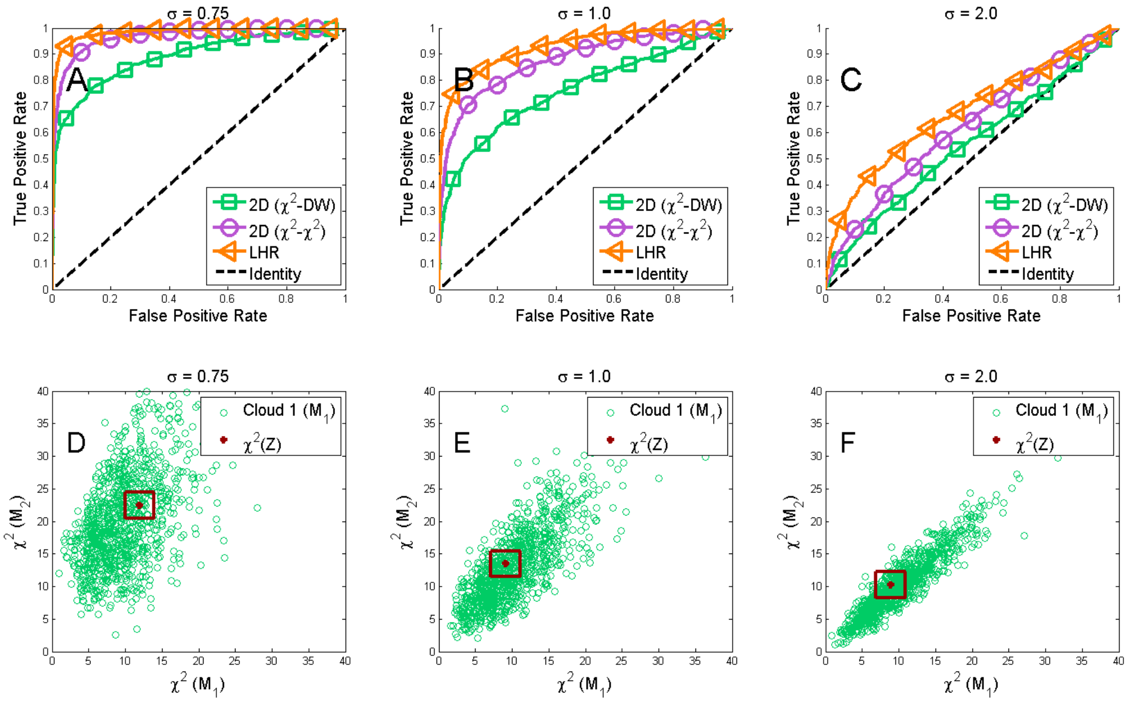

Supplementary Figure S8: The effect of varying the noise level on ROC curves and some example clouds. (A,B,C) ROC curves for varying degrees of noise used in the data generating process, using σ= 0.75 (A), σ=1 (B), and σ=2 (C). ROC curves are included for 2D χ^2 vs DW (green squares), 2D χ^2 vs χ^2 (purple circles) and LHR (orange triangles). (D,E,F) Example χ^2 vs χ^2 clouds corresponding to the 2D χ^2 vs χ^2 method for the same levels of noise as (A,B,C) respectively. When noise levels are increased, the false model becomes more adept at describing data generated by the true model. This means that it will be harder to discriminate between the two models and this is reflected in the fact the the ROC curves approach the identity line for higher levels of noise. However, the ordering of the methods with respect to AUC is still the same and does not vary with noise levels. The problem of discriminating between the models for increasing levels of noise can also bee seen in the different clouds. In (D), $\mathcal{M}_1$ (here the true model) has lower costs than $\mathcal{M}_2$ (here the false model), on average. In (E) and (F) the trend towards equal ability of the models to describe new data is shown: the clouds lie increasingly along the 45 degree diagonal.
